# Trichostatin A Modulates Ethanol Consumption and Reveals Dose- and Sex-Specific Transcriptomic Signatures in the Nucleus Accumbens Shell

**DOI:** 10.3390/cells15161494

**Published:** 2026-08-19

**Authors:** Yi Zou, Sheketha R. Hauser, Teresa J. Raba, Richard L. Bell, Zhao Lai, Tiebing Liang

**Affiliations:** 1 Greehey Children’s Cancer Research Institute, UT Health San Antonio, San Antonio, TX 78229, USA; zou@uthscsa.edu (Y.Z.); laiz@uthscsa.edu (Z.L.); 2 Department of Psychiatry, Indiana University School of Medicine, Indianapolis, IN 46202, USA; shhauser@iu.edu (S.R.H.); ribell@iu.edu (R.L.B.); 3 Paul and Carole Stark Neurosciences Research Institute, Indiana University School of Medicine, Indianapolis, IN 46202, USA; 4 Department of Medicine, Indiana University School of Medicine, Indianapolis, IN 46202, USA; traba@iu.edu; 5 Department of Molecular Medicine, UT Health San Antonio, San Antonio, TX 78229, USA

**Keywords:** high alcohol drinking-1 rats, Trichostatin-A, epigenetics, sex difference, nucleus accumbens shell, RNA-seq

## Abstract

**Background**: Histone deacetylase inhibitors (HDACis) such as Trichostatin A (TSA) have emerged as promising epigenetic modulators of addiction-related behaviors. TSA treatment has been previously shown to decrease alcohol (ethanol) consumption with dose and sex differences. However, molecular mechanisms underlying TSA’s effects on ethanol consumption remain poorly understood. **Methods**: We collected the nucleus accumbens shell (NAcSh) of HAD1 rats, which has been used to investigate the impact of TSA treatment on ethanol consumption. RNA-seq profiling of NAcSh followed by IPA and GSEA analysis were conducted. **Results**: Gene profiling identified differentially expressed genes (DEGs) with sex- and dose-specific effects, with some genes demonstrating high fold change (FC). Males showed significantly increased *Oxt* and decreased *Ttr* expression following 1 mg/kg TSA treatment. In females, *Pmch* expression significantly decreased following 1 mg/kg TSA treatment, whereas *Tmem179* expression increased following 2 mg/kg TSA treatment. Pathways centered on *Hdac* and *Fkbp5* in males, and *Bdnf* and estrogen receptor in females were significantly enriched following TSA treatment, with distinct pathways identified at the 1 mg/kg and 2 mg/kg doses. IL1β and β-estradiol are common upstream regulators among all groups. Unexpectedly, some common DEGs between male and female comparisons have opposite responses to the same dose of TSA. GSEA analysis has identified additional gene sets, hallmark genes and microRNAs, and functions including immune response, metabolism, and estrogen response significantly associated with TSA treatment. **Conclusions**: This study successfully identified gene expression evidence that TSA treatment is sex- and dose-specific, underscoring the importance of considering both variables in the development of HDAC-targeted therapies for alcohol use disorders.

## 1. Introduction

Alcohol use disorders (AUDs) are characterized as a chronically relapsing condition and continuation of alcohol use despite negative consequences. Chronic use of alcohol can cause or exacerbate health problems, increase mortality rates, and cause social problems. The cost to the US economy is roughly USD 250 billion per year and has been further intensified by the COVID-19 pandemic [1,2,3,4]. The heritability of AUD is about 50–60% of the total phenotypic variability [5,6,7,8]. The “missing heritability” is from environmental factors, epigenetic factors, and their interactions [9,10,11]. Genetic predisposition is critical for AUD development, but irreversible; epigenetic modifications occur over time by alcohol exposure and are reversible [12,13,14]. Effective treatments for AUD are limited, and there is a pressing need for new and effective medications to treat AUD [15].

Researchers recognize that alcohol (ethanol)-induced gene expression alterations are partially due to epigenetic modifications [13,16]. Changing chromatin density prepares it for transcriptional activity, resulting in gene expression alterations in response to ethanol exposure [17,18,19]. In the brains of AUD patients, ethanol has been shown to induce histone-related modification, DNA methylation, and other epigenetic changes in several brain reward systems [11,20,21], and converging evidence suggests that these modifications play roles in ethanol-induced gene regulation and behavior alteration [22,23,24,25]. Genes implicated in ethanol drinking have also been modified epigenetically (e.g., brain-derived neurotrophic factor (*BDNF)*, synuclein alpha (*SNCA)*, and nuclear receptor subfamily 3 group C member 1 (*NR3C1*)) [13,23,26]. Epigenetic regulation of neurotransmission-related genes, including *NR2B*, and *OPRM1*, plays a critical role in ethanol dependence and withdrawal, and predicts naltrexone treatment response [27,28]. Acetylation/deacetylation of histones is facilitated by a large family of histone acetyltransferases (HATs) and histone deacetylases (HDACs). Gene expression levels and protein activity of HDACs can be increased by chronic ethanol exposure [29]. For example, increased expression of *Hdac2* and *Hdac3* was observed after chronic ethanol exposure [19]. Co-exposure to HDAC inhibitors (HDACi) counteracted ethanol-induced transcriptional alterations; one study found that Trichostatin A (TSA; an HDACi) prevented chronic ethanol-induced suppression of the GABAA receptor α1 subunit gene (Gabra1) through reversal of HDAC-mediated chromatin remodeling [19]. Other evidence from the nucleus accumbens (NAc) also supports that excessive alcohol drinking reduces histone H4 acetylation, and exposure to HDAC inhibitors decreased ethanol consumption in rodents [30]. Antisense-induced downregulation of circadian genes attenuates ethanol-induced increases in HDAC2 in the medial nucleus accumbens shell (NAcSh) and significantly reduces alcohol consumption [31]. Researchers also found epigenetic differences in a sex-specific manner [32,33]. Currently, the impact of ethanol exposure on epigenetic modification has become a direction of interest for new drug development [30,34].

HDACis have emerged as promising therapeutic agents for several diseases, including AUD. TSA, a first-generation pan-HDAC inhibitor and prototype compound that led to the development of the FDA-approved HDAC inhibitor *Vorinostat*, has been widely used to investigate the role of epigenetic regulation in AUD. TSA was selected because chronic ethanol exposure increases HDAC activity and disrupts histone acetylation, leading to maladaptive transcriptional changes associated with ethanol dependence, anxiety-like behavior, and excessive drinking in preclinical research. Studies have shown that ethanol upregulates HDAC2 through oxidative stress pathways, whereas TSA reverses these changes by reducing HDAC expression, restoring histone acetylation, and normalizing gene transcription [35,36]. TSA also attenuates ethanol-induced alterations in class I HDACs, modulates neuroimmune signaling through effects on cytokines, chemokines, and NF-κB activity, and prevents ethanol-related disruptions in GABAergic neurotransmission [19,35,37,38]. Consistent with these mechanisms, TSA reduces ethanol consumption and anxiety-like behaviors in multiple rodent models, supporting a critical role for epigenetic regulation in AUD pathogenesis and treatment. Collectively, these findings suggest that HDAC inhibition may represent a promising therapeutic strategy for AUD. Currently, research has implicated HDAC activity in the development of ethanol dependence and ethanol-seeking behaviors, suggesting that TSA inhibition of HDACs can be a therapeutic approach [13].

The brain processes ethanol drinking behavior through distributed neural circuits and genetic, epigenetic, and environmental factors that collectively affect AUD development. However, investigation of drug effects in the human brain is limited by experimental and ethical constraints. The high alcohol drinking (HAD) replicate line of rats satisfies the criteria for an animal model of alcoholism [39,40,41]. HDACis have been reported to reduce ethanol drinking in male HAD1 rats [42]. In a subsequent experiment, our co-authors examined the TSA dose response in chronic ethanol-drinking models, and their findings on TSA’s effects on ethanol consumption in HAD1 rats align with earlier research [43].

The NAcSh is a key component of the mesolimbic reward circuitry that regulates ethanol consumption through motivational and emotional processes and neuron adaptations, and deep brain stimulation to the NAcSh or NAc core significantly reduced ethanol intake in rats [44,45,46]. Epigenetic changes and neuronal excitability adaptations in the NAc caused by chronic ethanol consumption are associated with molecular remodeling that promotes excessive ethanol consumption [47]. A previous study found binge ethanol drinking results in divergent gene and pathway alterations between sexes in the NAc of C57/6J mice [48].

Pharmacologic intervention followed by transcriptome profiling will provide transcriptomic evidence of potential epigenetic mechanisms and their involvement in AUD, helping to inform future drug development. Therefore, the objectives of the present study were to evaluate gene expression alterations in the NAcSh after 1 mg/kg/day and 2 mg/kg/day TSA treatment using the same rats mentioned above, and to identify gene expression and associated pathway alterations in HAD1 rats responsible for the observed decrease in drinking between sexes and doses.

## 2. Materials and Methods

### 2.1. Test Compound and Treatment Procedures

Trichostatin A (TSA; Sigma-Aldrich, St. Louis, MO, USA) administration has been performed previously [43]. In short, adult male and female HAD1 rats were given continuous 24 h 3-bottle free-choice access to 15%, 30% ethanol vs. water for at least 8 weeks (Figure S1). Food and water were available ad libitum. The 9th week of access served as the baseline week, such that the animal dose groupings were balanced for the average daily ethanol intake. After the baseline week, the animals underwent five consecutive days of testing (M–F), with each rat receiving its respective dose once daily. During the test week, TSA was administered intraperitoneally (i.p.) at 1.0 or 2.0 mg/kg doses. These doses were selected to approximate those previously reported to reduce ethanol drinking in P rats [49].

### 2.2. RNA Isolation

NAcSh tissue samples were obtained from 49 rats [male controls (*N* = 8), 1.0 mg/kg (*N* = 8), and 2.0 mg/kg (*N* = 8); female controls (*N* = 8), 1.0 mg/kg (*N* = 8), and 2.0 mg/kg (*N* = 9)]. In short, brains were snap-frozen and stored at −80 degrees. NAcSh tissue was collected by micro-punch, with detailed methodology outlined in our previous publication [50]. RNA was isolated using a Macherey-Nagle Nucleospin RNA isolation kit. All samples had a high yield, and the average total amount of RNA of each group ranged from 300 ng to 470 ng. The quality of RNA was measured by nanodrop and bioanalyzer. There was no significant difference in RNA quality between groups, with the average RNA Integrity Number (RIN) range of each group being 8.7–9.1.

### 2.3. RNA-Seq Profiling and Data Analysis

RNA-seq was performed using the Illumina TruSeq RNA sample preparation protocol. The resulting libraries were sequenced on an Illumina HiSeq 2000 instrument using a standard single-end 50 bp sequencing protocol. The reads were aligned to the reference *Rattus norvegicus* genome (UCSC Rn 6.0) with TopHat 2 [51]. No more than 2 mismatches were allowed in the alignment. HTseq was used to count gene expression (reads), and DEseq was used to find differentially expressed genes (DEGs) after performing median normalization [52,53]. Significant DEGs were identified with adjusted *p* < 0.05 for multiple tests by the Benjamini–Hochberg method for controlling false discovery rate [54] (gene has read abundance with RPKM ≥ 1 within at least one treatment [sample]) to ensure only high-confidence transcripts were analyzed. Pairwise differential gene expression (DGE) analysis was performed using the DESeq package in R based on raw read counts. Downstream gene expression quantification and visualization were conducted using normalized RPKM values. Dose effect was assessed by comparing gene expression values between 2 mg/kg and 1 mg/kg, and presented as log_2_ ratio.

### 2.4. Ingenuity Pathway Analysis

Ingenuity Pathway Analysis (IPA) software (Content version: 60467501 and release date: 2020-11-19) was used to carry out bioinformatics analysis to identify canonical signaling pathways, upstream regulators, and downstream biological effects between control and treatment groups and between sexes. Causal network analysis allowed the discovery of regulatory networks for mechanistic understanding. *STRING* analysis (https://string-db.org/) identified interconnections between genes involved in the same function category. Cluster members with at least one connection with other members were included, and the line (edge) between two protein nodes represents a predicted or known functional or physical association between them.

### 2.5. Gene Set Enrichment Analysis (GSEA)

GSEA was performed to identify biologically relevant gene sets associated with TSA treatment dose [55,56]. The algorithm ranks all genes based on differential expressions and calculates an enrichment score (ES) for each gene set, reflecting its overrepresentation at the top or bottom of the ranked list. A positive ES indicates enrichment among upregulated genes, while a negative ES indicates enrichment among downregulated genes. Based on gene ranks by pairwise FC, GSEA (GSEA, Broad Institute GSEA v4.3.3) was performed using a pre-ranked gene list generated from pairwise differential expression analysis, with genes ranked by log_2_ FC. (https://www.gsea-msigdb.org/gsea/index.jsp, and GSEA analysis date: 24 March 2021). Furthermore, we identified hallmark gene sets, which were collected from the Molecular Signatures Database (MSigDB) (h.all.v7.3.symbols.gmt) and the Human_RefSeq_Accession_MSigDB.v7.3.chip annotation file [57]. All other parameters were set to their default values. Gene sets with FDR *q*-value < 0.25 were considered significantly enriched, and the top enriched pathways were selected for downstream interpretation and visualization.

## 3. Results

### 3.1. Gene Expression Profiling Revealed Dose- and Sex-Specific TSA Responses

The NAcSh samples were obtained from animals used in a previously published study [43]. TSA treatment (1 and 2 mg/kg) consistently reduced ethanol intake in male HAD1 rats at both 4 h and 24 h time points. In females, TSA (1 and 2 mg/kg) was more effective at reducing ethanol intake at the 4 h than at the 24 h time point.

**Figure 1:**
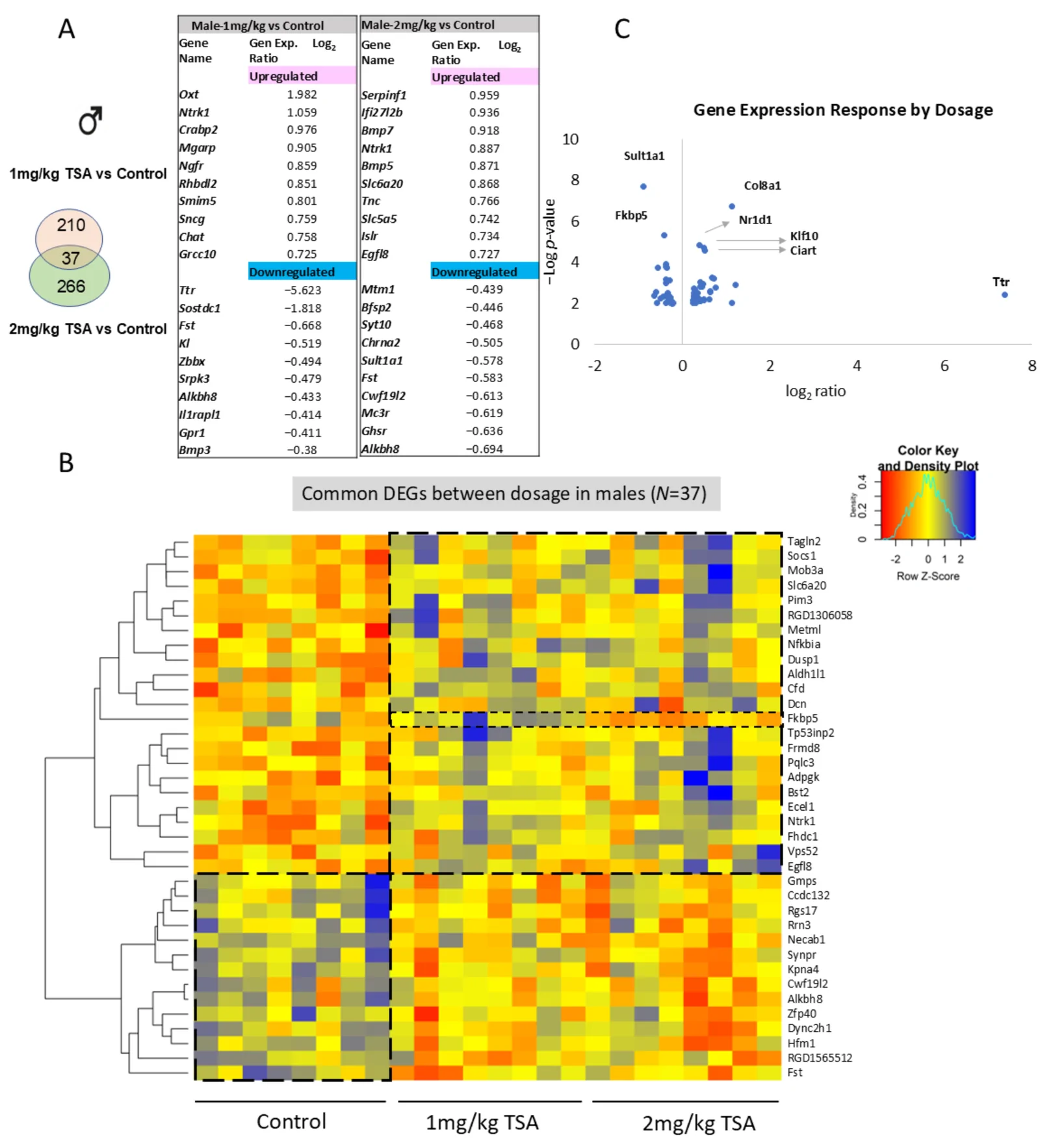
Differentially expressed gene (DEG) identification in males. (**A**) A Venn diagram depicts the number of genes that were differentially expressed compared to the control after 1 mg/kg and 2 mg/kg TSA dose in NAcSh of male HAD1 rats. The numbers of genes in common are represented in the overlapping section of the diagram. The top 10 upregulated and downregulated genes in males at 1 mg/kg and 2 mg/kg doses of TSA are listed, along with the expression FC by Log_2_ ratio for each gene. Upregulated genes compared to the control are indicated in pink, and downregulated genes are indicated in blue, and the “−“ value indicates downregulated Log_2_ ratio. (**B**) The heatmap displays dose-responsive DEGs that are in common between doses (N = 37) and their clustering pattern with increasing dose in NAcSh of male HAD1 rats. (**C**) Gene expression response by dose. Fountain plots depict the gene expression alterations by dose and their significance. A positive value indicates increased gene expression when increased from 1 mg/kg to 2 mg/kg dose; on the contrary, a negative value indicates decreased gene expression after increasing dose. Some of the genes with higher FC ratio between 2 mg/kg and 1 mg/kg and significant *p*-values were labeled. In the heatmap, the color key indicates row Z-score, and the light blue line represents the kernel density estimate of the distribution of row-scaled expression values.

RNA-seq analysis identified differentially expressed genes (DEGs) between control and dose treatments using a two-level *p*-value cutoff (*p* < 0.05 and *p* < 0.01) and various FC cutoffs (Table S1). The analysis provides a broad overview of dose effects, and a majority of DEGs have small FC. Figure 1A shows that in male rats, a similar number of dose-specific DEGs were identified when comparing the 2 mg/kg dose to the control group (N = 266) and 1 mg/kg dose to the control group (N = 210) (*p* < 0.05, FC > 1); a majority of DEGs were found to be dose-specific, while 37 DEGs were found to be common between the two doses. The top 10 upregulated and downregulated DEGs at each dose treatment reveal distinct gene expression profiles, and it was noted that a few upregulated genes had a much higher FC by log_2_ ratio (e.g., oxytocin (*Oxt*) and neurotrophic receptor tyrosine kinase 1 (*Ntrk1*)) ratios were higher than the rest, as well as a few downregulated genes (e.g., transthyretin (*Ttr*) after 1 mg/kg TSA treatment) (Figure 1A).

Gene dose response is an important pharmacological aspect for drug development. To understand the DEGs shared between doses (N = 37), we performed clustering analysis and identified clusters of genes with increased or decreased expression as dose increased compared with the control group. A heatmap depicts an overview of upregulated and downregulated gene clusters with increasing dose, with an exception (e.g., *Fkbp5* has a different dose response) (Figure 1B). Expanding to all DEGs, we analyzed genes that are positively or negatively correlated with increasing TSA dose. The gene expression ratio between 2 mg/kg and 1 mg/kg TSA was calculated, and a fountain plot allowed for visualization of their significance by *p*-value and log_2_ ratio between doses (Figure 1C). When plotting dose DEGs with *p* < 0.01 and FC > 1 cutoff, 41 vs. 26 were positively or negatively correlated with increasing dose, respectively. Some genes with a more significant *p*-value or a higher ratio are labeled on the plot. For example, *Ttr* has the highest increased gene expression ratio, and *Col8a1* has increased gene expression with the highest *p*-value in males (Figure 1C); *Sult1a1* and *Fkbp5*, both epigenetically modifiable genes, have the most significant *p*-value and are negatively correlated with increasing dose in males (Figure 1C). The top 20 most significant genes by *p*-value, along with their expression levels, FC, and their correlation with increasing dose, are presented in Table 1.

**Figure 2:**
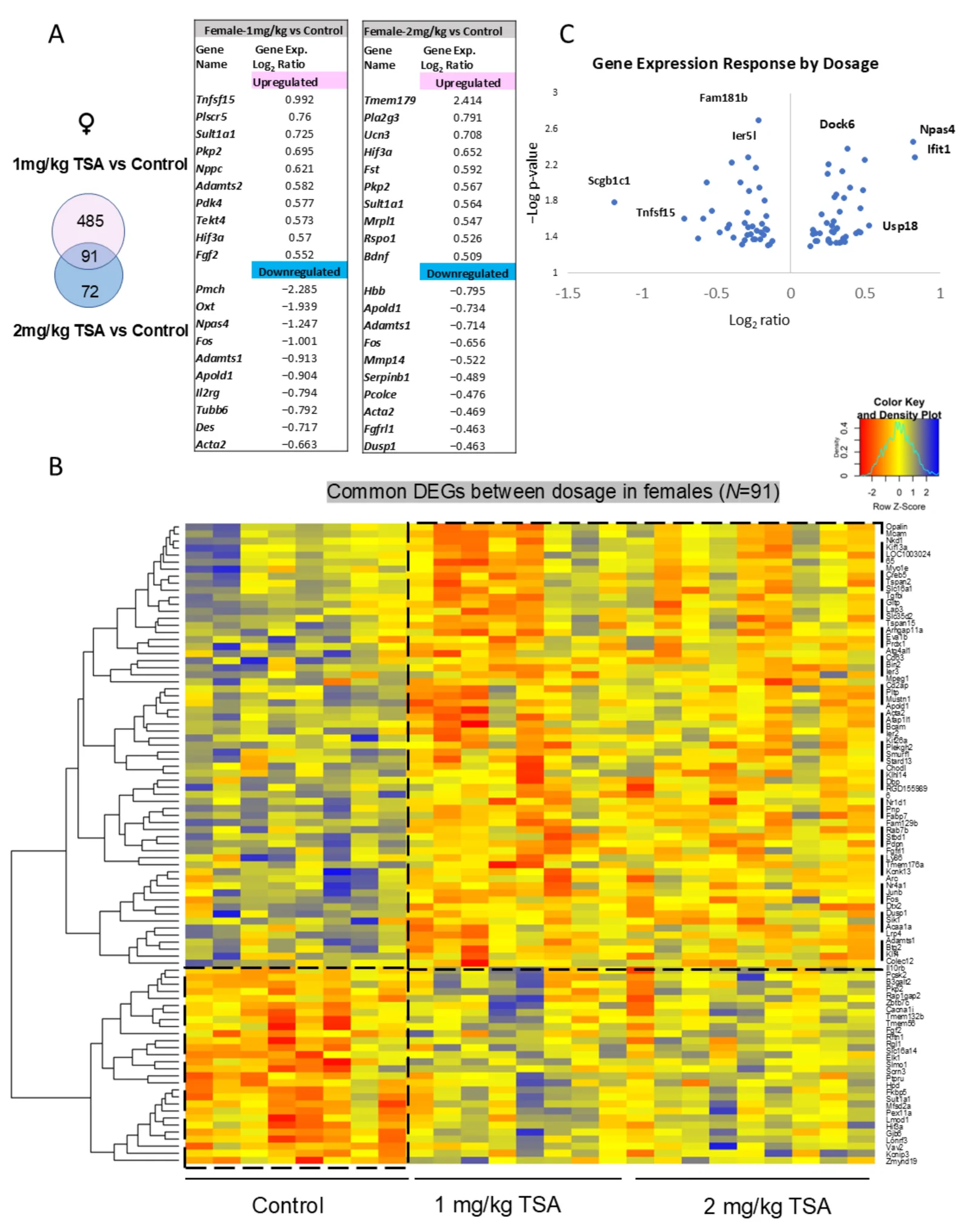
Differentially expressed gene (DEG) identification in females. (**A**) A Venn diagram depicts the number of genes that were differentially expressed compared to the control after 1 mg/kg and 2 mg/kg TSA dose in NAcSh female HAD1 rats. The number of genes in common are represented in the overlapping section of the diagram. The top 10 upregulated and downregulated genes in female rats at 1 mg/kg and 2 mg/kg doses of TSA are listed along with the expression FC by Log_2_ ratio for each gene. Upregulated genes compared to the control are indicated in pink, and downregulated genes are indicated in blue, and the “−“value indicates downregulated Log_2_ ratio. (**B**) The heatmap displays dose-responsive DEGs that are in common between doses (N = 91) and their clustering pattern with increasing dose in NAcSh of female HAD1 rats. (**C**) Gene expression response by dose. Fountain plots depict the gene expression alterations by dose and their significance. A positive value indicates increased gene expression when increased from 1 mg/kg to 2 mg/kg dose; on the contrary, a negative value indicates decreased gene expression after increasing dose. Some of the genes with higher FC ratios between 2 mg/kg and 1 mg/kg and significant *p*-values were labeled. In the heatmap, the color key indicates row Z-score, and the light blue line represents the kernel density estimate of the distribution of row-scaled expression values.

In female rats, the number of dose-specific DEGs varied significantly, with the 1 mg/kg dose resulting in more than six times as many DEGs as the 2 mg/kg dose (485 vs. 72, respectively). Additionally, 91 DEGs were in common between the 1 mg/kg and 2 mg/kg dose groups (Figure 2A). The top 10 upregulated and downregulated DEGs at each dose level revealed distinct gene expression profiles, with some genes exhibiting higher FC. Pro-melanin concentrating hormone (*Pmch*), oxytocin/neurophysin I prepropeptide (*Oxt*), and neuronal PAS domain protein 4 (*Npas4*) were downregulated after 1 mg/kg TSA treatment in females. Transmembrane protein 179 (*Tmem179)* increased significantly after 2 mg/kg TSA treatment (Figure 2A). Similarly, in females, to understand the DEGs that are in common (N = 91) between doses, we performed clustering analysis and grouped genes that were upregulated or downregulated in response to increasing dose (Figure 2B). From all identified DEGs in females, some genes that had the most significant *p*-value were positively correlated with treatment dose, like interferon-induced protein with tetratricopeptide repeats 1 (*Ifit1*), neuronal PAS domain protein 4 (*Npas4*), and dedicator of cytokinesis 6 (*Dock6*) (Table 1); genes negatively correlated with increasing dose treatment included family with sequence similarity 181 and 134b, member B (*Fam181b* and *Fam134b*) and immediate early response 5-like (*Ier5l*) (Table 1). A fountain plot (Figure 2C) is included for visualization of the significance and FC of DEGs in females (*p* < 0.01 and FC > 1). Therefore, the DEG profile revealed a dose-specific response to TSA treatment, with fewer DEGs shared across doses.

### 3.2. Common DEGs Between Sexes Respond Divergently Within the Same Dose

**Figure 3:**
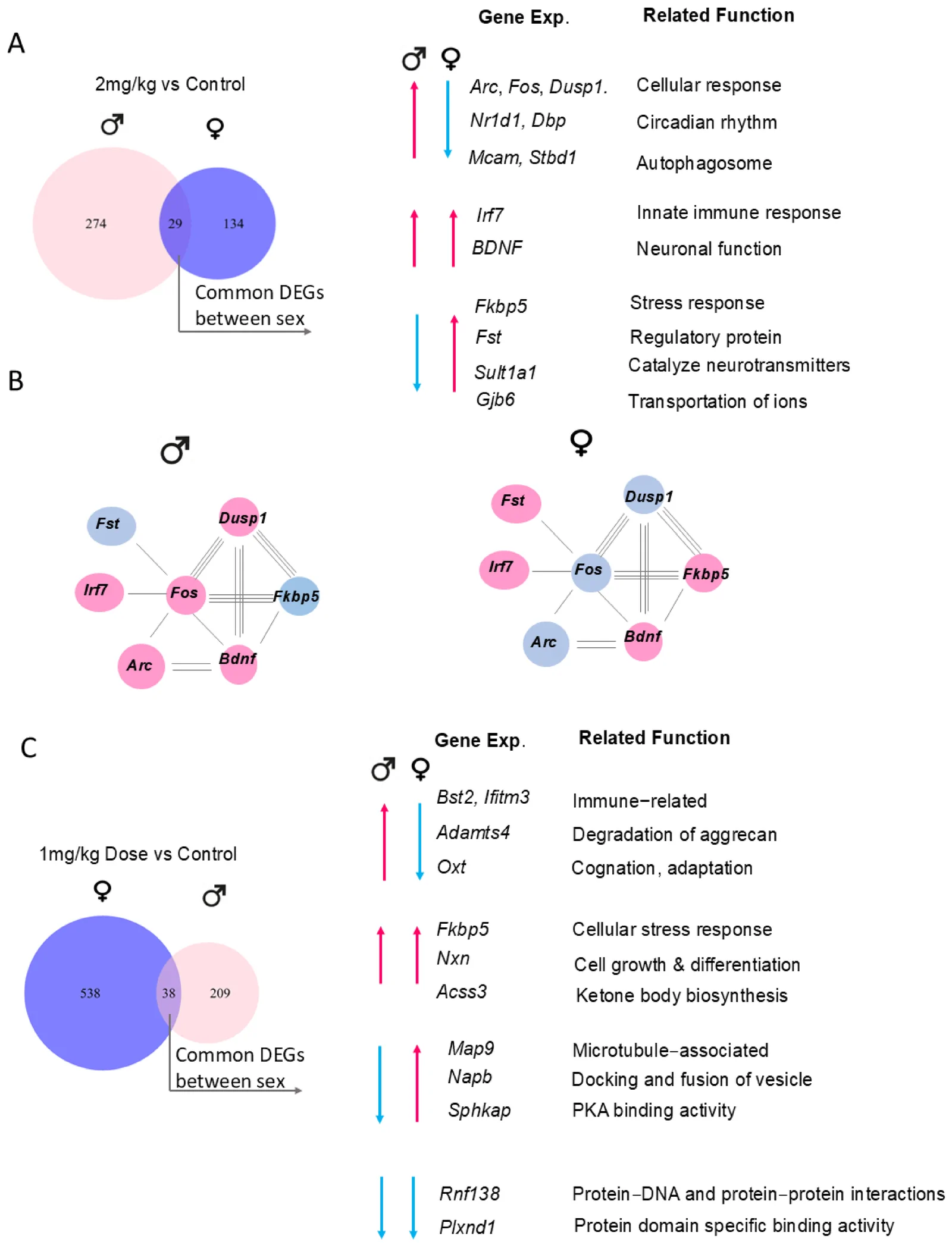
Sex-specific and in common DEGs after the same dose treatment. (**A**) A Venn diagram shows the number of differentially expressed genes in NAcSh of male and female HAD1 rats after administration of a dose of TSA compared with the control group. The overlapping section of the Venn diagram shows the number of genes that are shared in male and female rats administered a 2 mg/kg dose of TSA. The right panel shows genes of interest, their associated functions, and the relationship between their expression in males and females after 2 mg/kg TSA treatment. (**B**) *STRING* analysis prediction of protein–protein interaction (PPI) network that connects *Fos* gene in males and females. Blue nodes indicate decreased gene expression, and pink nodes indicate increased expression. Red arrow indicates increased, and blue arrow decreased, gene expression following TSA treatment. The line (edge) in between the gene nodes represents a predicted or known functional or physical association between them. (**C**) A Venn diagram shows the number of differentially expressed genes in male and female HAD1 rats after administration of a 1 mg/kg dose of TSA compared to the control group. The overlapping section of the Venn diagram shows the number of genes that are shared in male and female rats administered a 1 mg/kg dose of TSA. The right panel shows genes of interest, their associated functions, and the relationship between their expression in males and females after 1 mg/kg TSA treatment.

Further analysis of the sex difference within the same dose showed that DEGs are sex-specific in response to TSA, and only a small number of DEGs were shared between sexes when given the same dose of TSA (Figure 3). We focused on analyzing those DEGs that were in common between sexes after the same dose treatments; surprisingly, among 29 DEGs in common, only Interferon regulatory factor 7 (*Irf7*) and *Bdnf* increased in both males and females after 2 mg/kg treatment (Figure 3A); 27 genes responded in opposite directions (Table 2). We expect that these genes and their functions could be differentially regulated and may lead to different behavioral consequences (Figure 3A). For example, activity regulated cytoskeleton associated protein (*Arc*) and *Fos* are immediate early genes, critical for early response and pathology of neurological diseases [50,58]. *Fos* is in a hub with multiple genes and regulates cellular response and immune response (Figure 3B). However, they respond in opposite directions in males and females. Dimorphic regulation of Fos was also found in a mouse binge ethanol drinking model [48]. Dual specificity phosphatase 1 (*Dusp1*) plays an important role in cellular and environmental stress, including ethanol [59,60]. *Fkbp5* and *Bdnf* are both linked to stress response and cognition and are epigenetically modifiable [61,62]. These genes work in concert to regulate cellular functions in both sexes, but divergent gene expression profiles might lead to different functional alterations. Similarly, we also found that among 38 genes in common between sexes after 1 mg/kg treatment, *Fkbp5* and others have the same increased expression, while *Plxnd1* and others have the same decreased expression in both sexes; however, more genes have an opposite trend of response (e.g., *Oxt*) (Table 2). The functions of some of these genes are highlighted in Figure 3C. This observation further suggests that gene expression response to TSA is sex-specific.

### 3.3. Pathways Associated with Neuropathic Pain Signaling, Inflammation, and Stress Response Were Altered After TSA Treatment

**Figure 4:**
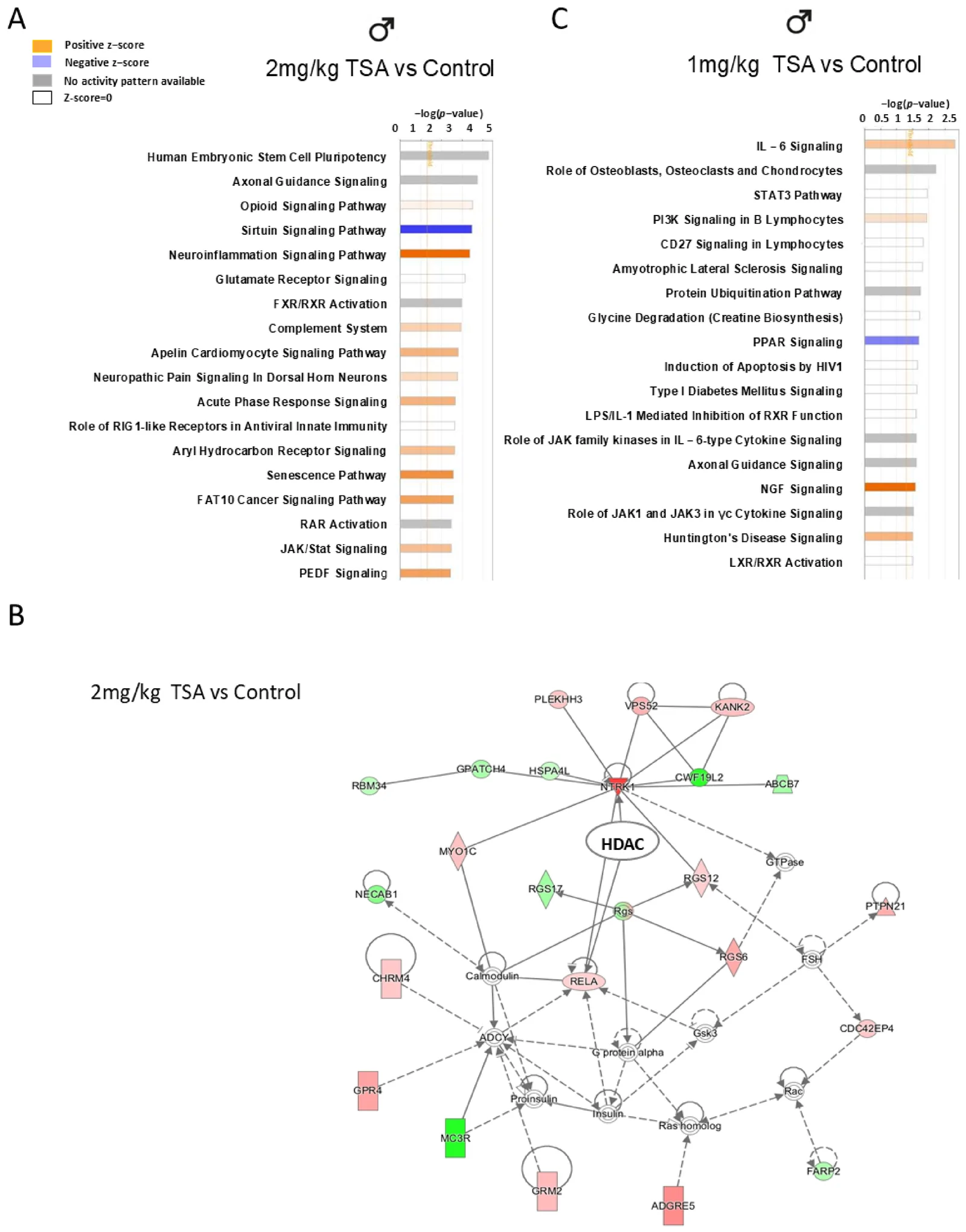
IPA analysis of significant pathways by different TSA treatment doses in males. (**A**) The most significant pathways in male HAD1 rats that were given a 2 mg/kg dose of TSA. An orange bar is indicative of a positive z-score and increased activity, and a blue bar represents a negative z-score and a decrease in pathway activity. A gray bar indicates no activity pattern available, and a white bar indicates a z-score of 0 and no change in pathway activity compared to the control. (**B**) HDAC-centered IPA pathway after 2 mg/kg dose of TSA. (**C**) The most significant pathways in male HAD1 rats that were given a 1 mg/kg dose of TSA. Red indicates increasing, and green decreasing, gene expression. Solid lines indicate direct physical interaction, and dashed lines indicate two molecules affecting each other through one or more intermediate or regulatory steps.

To understand the involvement of these DEGs in pathways, they were used as input for Ingenuity Pathway Analysis (IPA) to predict relevant molecular networks, biological functions, and canonical pathways. The most significant pathways were identified based on activation z-scores and statistical significance (*p*-values). The top IPA pathways in males given 2 mg/kg treatment (Figure 4A) were identified when compared to controls. Negative regulation of the Sirtuin signaling pathway and positive regulation of neuroinflammation were among significant IPA pathways when comparing 2 mg/kg dose with the control (Figure 4A). The primary activity of sirtuin is deacetylation, and it plays a key role in addiction-related brain changes. Sirtuin signaling, involved in senescence and aging, plays a role in metabolic and stress response pathways [63,64,65]. A canonical pathway identified in 2 mg/kg treatment is centered around *Hdac* and interacts with upregulated neurotrophic receptor tyrosine kinase 1 (*Ntrk1*) and RELA proto-oncogene, NF-kB subunit (*Rela*, also known as p^65^), highlighted in Figure 4B. Consistently, another pathway including upregulation of NF-kB complex centered around IRF7 suggests de-repression of master transcription factor response for innate immune response; downregulation of cholinergic signaling (nAchR, CHRNA2) for mediating “dampening” of some receptors. The presence of HAT (Histone Acetyltransferase) and NAP1L2 (Nucleosome Assembly Protein) showed that TSA’s inhibition of HDACs actively shifted the chromatin toward an open state, particularly loci that control immune vigilance (Figure S2A).

When comparing 1 mg/kg vs. control in males (Figure 4C), some pathways were activated, including IL-6 signaling, NGF-signaling, and Huntington’s disease signaling, while PPAR signaling was inactivated. Among them, one worth mentioning is the pathway centered around the downregulation of transthyretin (*Ttr*) and ubiquitin protein ligase E3 component n-recognin 1 (*Ubr1*), but upregulation of *Fkbp5* and *Ntrk1* (Figure S2B). Another hub gene in this pathway is a master regulator, Fragile X Messenger Ribonucleoprotein 1 (FMR1), which plays a role in regulating synaptic plasticity and involves mRNA transportation and regulation in neurons (Figure S2B). Further IPA causal network analysis also indicated that glucocorticoid (GC) and its receptor (GR) are implicated in TSA treatment response.

To understand dose effect and functional alterations, we compared the impact of 2 mg/kg vs. 1 mg/kg TSA treatment in males. Differentially regulated genes (WNT3A and EGF) altered cell migration, and mesenchymal differentiation functions were identified, and this network is modulated by circadian (ARNTL), transcriptional (ZBTB16) and neuronal guidance pathways (Figure S3A). Likewise, pathways that are commonly regulated between doses in males, such as axonal guidance signaling, IL-6 signaling, Aryl Hydrocarbon Receptor Signaling (AHR) signaling, and more, are listed in Table 3. Overall, TSA treatment-associated epigenetic regulation affects pathways involved in transcriptional regulation, immune cell migration, axonal guidance, and cell communication.

**Figure 5:**
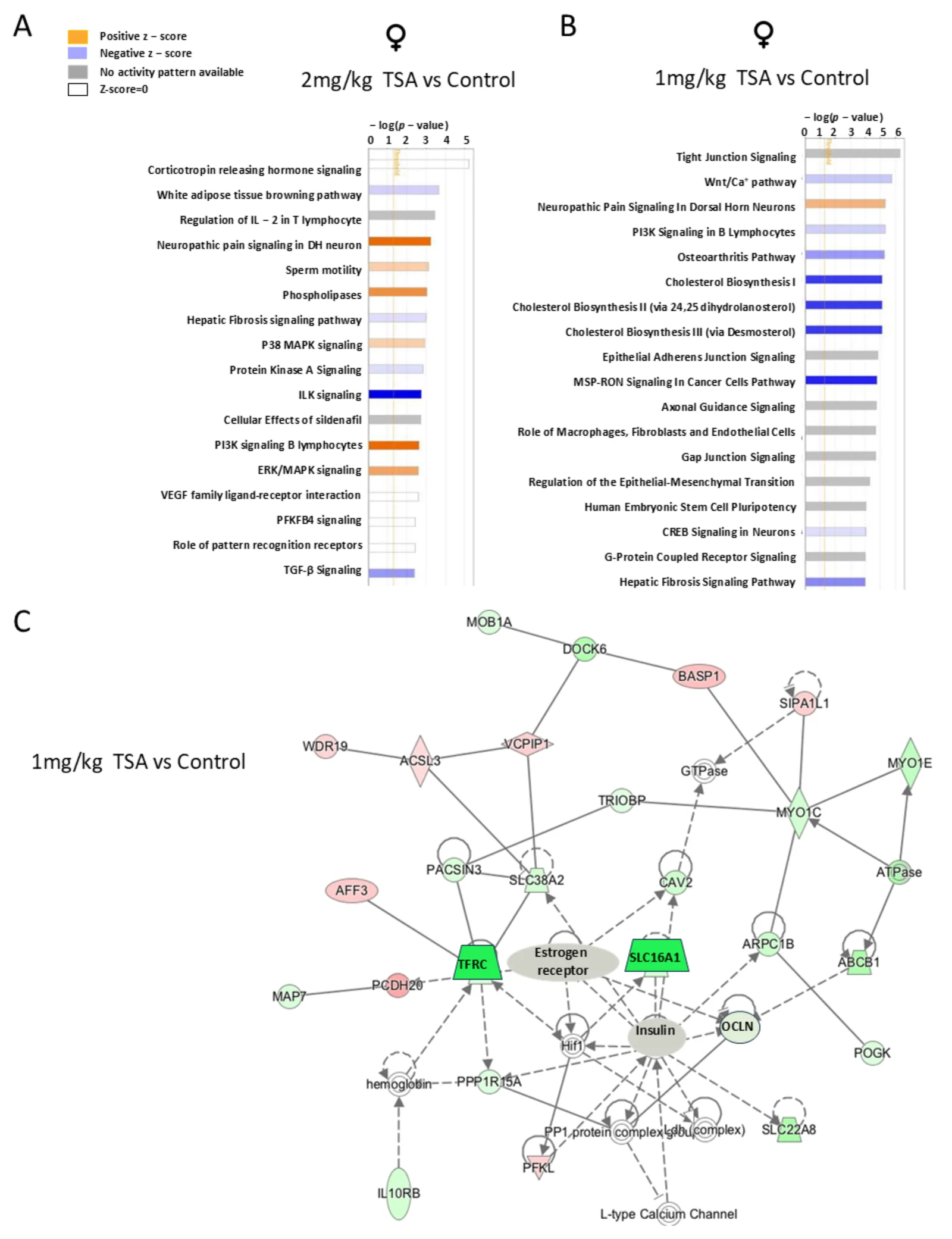
IPA analysis of significant pathways by different TSA treatment does in females. The most significant pathways in the NAcSh of female HAD1 rats that were given (**A**) a 2 mg/kg dose of TSA and (**B**) 1 mg/kg dose of TSA. An orange bar is indicative of a positive z-score and increased activity, a blue bar represents a negative z-score and a decrease in pathway activity, a gray bar indicates no activity pattern available, and a white bar indicates a z-score of 0 and no change in pathway activity compared to the control. (**C**) Pathway centered by estrogen receptor and insulin after 1 mg/kg dose of TSA. Red indicates increasing, and green decreasing, gene expression. Solid lines indicate direct physical interaction, and dashed lines indicate two molecules affecting each other through one or more intermediate or regulatory steps.

In females, top IPA pathways were identified by comparing 2 mg/kg TSA treatment to the control (Figure 5A) and 1 mg/kg dose to the control (Figure 5B), revealing multiple negatively regulated pathways that were not identified in male comparisons. Negative regulation of the white adipose tissue browning pathway, ILK signaling and TGF-β signaling was observed after 2 mg/kg TSA treatment (Figure 5A); negative regulation of multiple cholesterol biosynthesis-related pathways, Wnt/Ca^+^ pathway and PI3K signaling pathway, and CREB signaling in neurons after 1 mg/kg TSA treatment was observed (Figure 5B). The BDNF network was identified in females after 2 mg/kg TSA treatment. Upregulation of BDNF and *Plc* and downregulation of *Klf10*, *Nr4a1*, *Creb* and *TGFβ2* were identified and are highlighted in Figure S4A. BDNF has been found to regulate ethanol and cocaine use, and its expression is altered by epigenetic modification [14,66]. Analysis of the 1 mg/kg dose in females revealed alterations in metabolic pathways, especially the network centered around insulin that connects with *Slc16a1* and Occludin (OCLN). Additionally, both estrogen receptor and Transferrin Receptor 1 (TFRC1) play roles in lipid sensory, glucose homeostasis, and metabolically active neurons (Figure 5C). Another pathway involves Fkbp5, a GR binding protein that plays roles in ethanol consumption and withdrawal, as well as in response to stress and chemical stimulation [67,68,69]. Alteration of Fkbp5 affects neuronal development, RNA binding, and immune-related functions (Figure S4B). When comparing dose effect and response in females, the significance of inflammatory response and cell-to-cell signaling networks was observed. Overall, TSA treatment in females results in a coordinated immune response through signal transduction (STAT1, IRF7), cytokine signaling (IFN*γ*, *IL21*), and regulatory feedback (SIRT1 and TRIM24), as well as development of phagocyte processes (TLR4) (Figure S3B). Notably, a greater number of canonical pathways shared between the 2 mg/kg and 1 mg/kg doses were identified in females (see Table 3). Neuropathic pain signaling in dorsal horn neurons, corticotropin releasing hormone signaling, and regulation of IL-2 expression in activated and anergic T lymphocytes are among the top significantly altered pathways shared between doses in females. In summary, TSA influences genes involved in neuronal function, immune response, and metabolic processes, with regulatory effects that are distinctly sex- and dose-specific.

### 3.4. β-Estradiol Is a Shared Upstream Regulator Between Sexes and Doses

**Figure 6:**
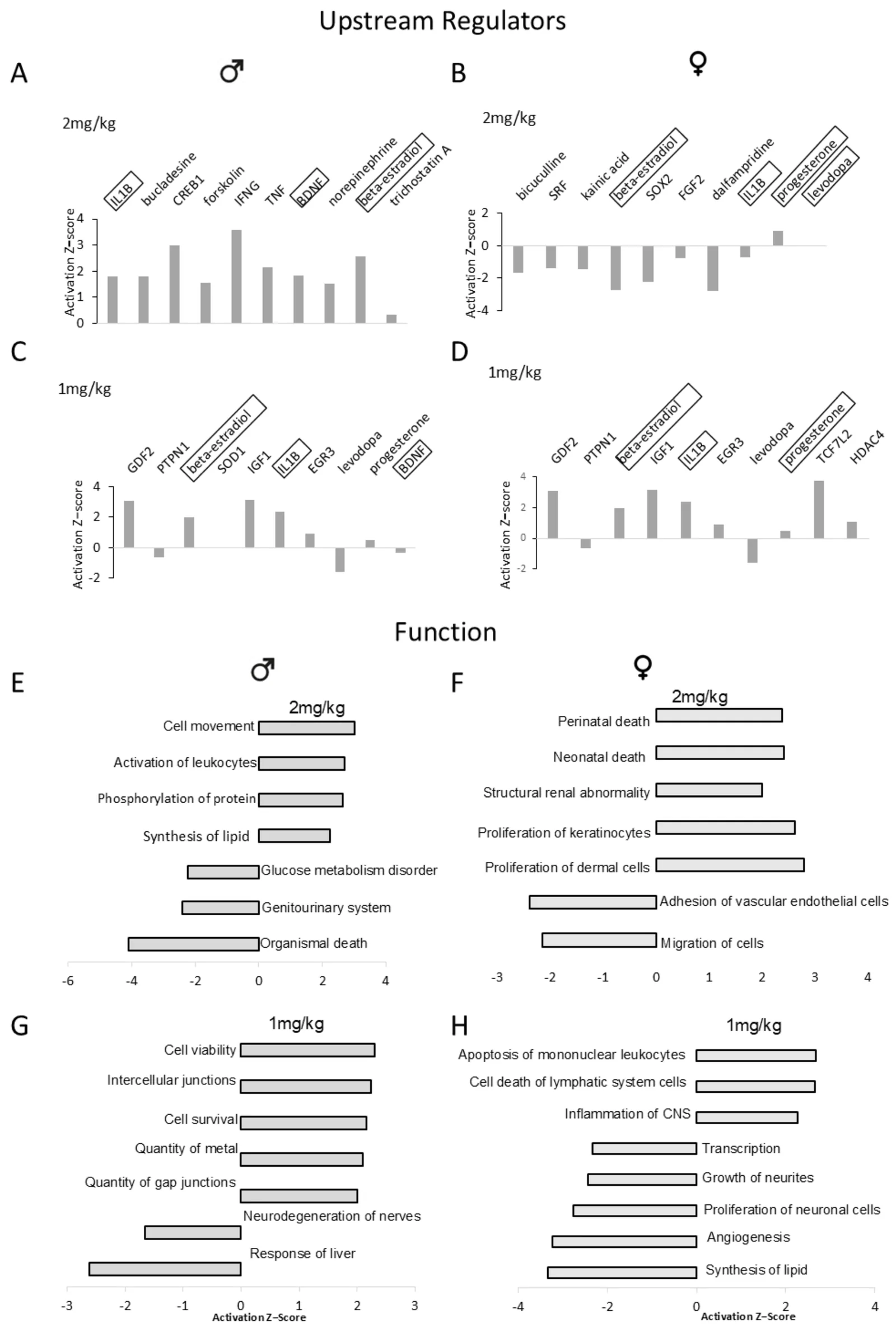
Analysis predicted upstream regulators that are activated or inhibited by TSA treatment. Predicted upstream regulators for (**A**) Male HAD1 rats and (**B**) Female HAD1 rats after 2 mg/kg dose TSA. (**C**) Male HAD1 rats and (**D**) Female HAD1 rats after 1 mg/kg dose TSA. Upstream regulators that are shared between sexes or doses are indicated with a black box. IPA analysis predicted disease functions that are affected by TSA treatment compared to the control group. (**E**) Male HAD1 rats and (**F**) Female HAD1 rats after 2 mg/kg dose TSA. (**G**) Male HAD1 rats and (**H**) Female HAD1 rats after 1 mg/kg dose TSA.

To understand the biological mechanisms driving the observed changes in gene expression, upstream regulator prediction brings the cause–effect relationship of TSA treatment response and directionality of regulation into focus. Figure 6A–D shows the top upstream regulators activated or inhibited by dose and sex. Some of these upstream regulators play a role in immune response (IL1B, IFNγ) and transcriptional regulation (CREB1, Sox2, TCF7L2, and SRF), as well as functioning as hormones (β-estradiol, progesterone), growth factors (IGF1), and many more. β-estradiol, the most potent and predominant form of estrogen that influences neurodevelopment, immune response, and gene expression, is predicted to be an upstream regulator of DEGs after TSA treatment.

Interestingly, β-estradiol and IL1B were identified as common top upstream regulators in all comparisons by dose in both sexes; however, it is inhibited in females after 2 mg/kg TSA treatment and activated in all other comparisons (Figure 6B). Other common upstream regulators were highlighted; again, BDNF is a common upstream regulator in males but with opposite activation z-score between doses (Figure 6A,C). Profound differences were found in females by dose, as most of the upstream regulators are inactive in 2 mg/kg TSA treatment but active in 1 mg/kg dose (except progesterone) (Figure 6B,D). The upstream regulator prediction generates hypotheses for future validation and potential drug targets.

The next question to address is which functions are altered after TSA treatment. IPA analysis has identified the most significant functions with predicted increased or decreased activation in males (Figure 6E,G) and in females (Figure 6F,H). In males, 2 mg/kg dose activation of cell movement and leukocytes indicates increased inflammatory response. Furthermore, phosphorylation of proteins is associated with activation of enzymes, and synthesis of lipids indicates increased metabolism. The 2 mg/kg dose also results in glucose metabolism and organismal death inactivation (Figure 6E). The 1 mg/kg dose activates cell viability, cell-to-cell signaling and interaction, and molecular transport in males (Figure 6G). In females, the 2 mg/kg dose increased perinatal and neonatal death and cellular growth and proliferation, while it inactivated migration of cells (Figure 6F). Effects of the 1 mg/kg dose in females included increased inflammatory response, decreased neuron dendritic growth, and neuron proliferation (Figure 6H). Gene function alteration consistently indicates that the 2 mg/kg dose may be less effective than the 1 mg/kg dose in females. The data suggest that dose effects on functional changes are divergent between sexes, with few commonalities.

### 3.5. GSEA Pathway Analysis

**Figure 7:**
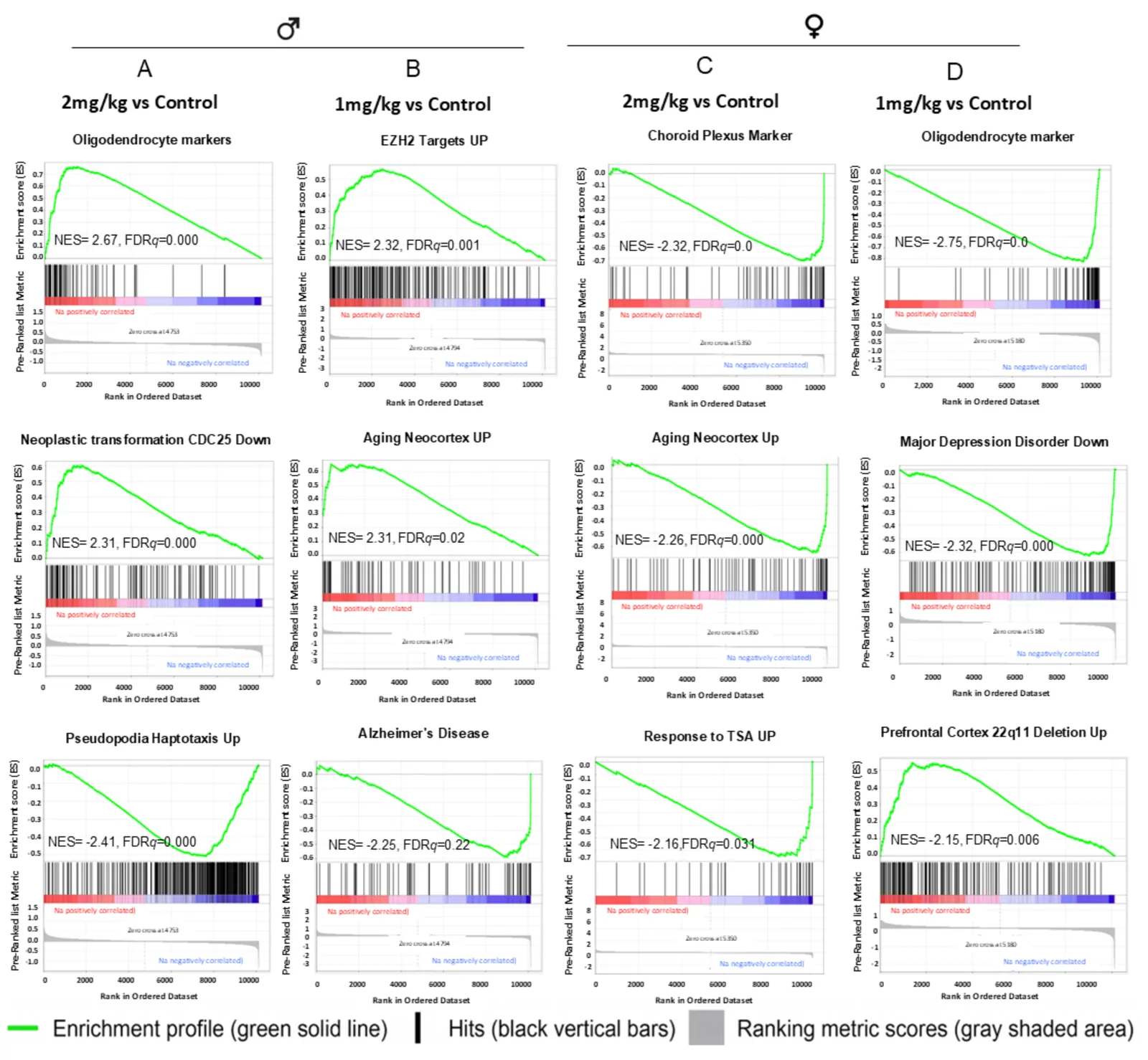
Representative gene set enrichment in each sex and treatment group. A positive normalized enrichment score (NES) indicates gene set enrichment at the top of the ranked list; a negative ES indicates gene set enrichment at the bottom of the ranked list. The green curve denotes the ES (enrichment score) curve, the running sum of the weighted enrichment score in GSEA. Normalized enrichment score (NES) and FDR *q*-value are indicated on the plot. (**A**) Male HAD1 rats after 2 mg/kg dose TSA and (**B**) Male HAD1 rats after 1 mg/kg dose TSA. (**C**) Female HAD1 rats after 2 mg/kg dose TSA and (**D**) Female HAD1 rats after 1 mg/kg dose TSA.

In addition to DEGs with relatively high FC, some other genes have low expression levels, but their pathway commonalities may contribute similarly to TSA treatment responses. In complement to DEG analysis, gene set enrichment analysis (GSEA) was performed to identify genes that were altered and enriched by treatment, as well as their effects on function. GSEA enrichment score curves plot the normalized enrichment score (NES) vs. significance (FDR *q*-value) of each gene set and determine specific gene set(s) that trend the top (Na-positive) or bottom (Na-negative) of the gene list, which is correlated with TSA treatment (Figure 7 and Figure S5). Comparing TSA treatment does with the control in both males and females, the bell-shaped plot indicates the enrichment score distribution, with the most significant ones in the yellow-colored area (FDR *q*-value < 25%, or nominal *p*-value < 0.05) (Figure S5A–D). Interestingly, more Na-positive enrichment gene sets were identified than Na-negative enrichment gene sets in both doses in males (Figure S5A,B); on the contrary, more Na-negative gene sets were identified in females of both dose treatments (Figure S5C,D). Table S2 summarizes the number of gene sets at various FDR cutoffs. These results from GSEA analysis not only support the results of the IPA analysis but also provide additional insights into different sex-specific responses to TSA treatment.

Table S3 lists the top identified gene sets in males and females. Some of the representative enrichment gene sets with enrichment profiles and ranking metric scores are presented (Figure 7A–D). In each gene set enrichment (GSE) profile plot, an upward peak or downward groove indicates enrichment of genes with positive Na scores or negative Na scores, respectively. For males given the 2 mg/kg dose, Figure 7A shows enrichment of oligodendrocyte markers and neoplastic transformation gene sets, indicating that TSA modulates oligodendrocyte-related gene expression, consistent with previously reported findings [70]. A pseudopodia haptotaxis gene set has Na-negative scores, indicating that TSA treatment suppresses expression of these genes and impairs cell motility [71]. Similarly, in 1 mg/kg TSA treatment in males, more Na-positive enrichment gene sets were found, and only one Na-negative gene set (Alzheimer’s Disease) was found (Figure S5B and Figure 7B). The oligodendrocyte marker gene set was also enriched in 1 mg/kg dose (Table S4), along with EZH2 targets and neocortex, suggesting altered gene expression associated with behavioral and neuronal deficits (Figure 7B) [72]. In the female comparison, more Na-negative gene sets were identified than Na-positive in both dose treatments (Figure S5C,D). Gene sets enriched in choroid plexus markers, aging, and response to TSA were identified (Table S4 and Figure 7C). Similarly, TSA treatment at 1 mg/kg in females results in the most significant enrichment of gene sets relevant to brain function (oligodendrocytes and dendrites) and to depression (Figure 7D). [73]. Few Na-positive gene sets were found, and the top ones were related to the prefrontal cortex, neurexins, and neuroligins. Of particular interest, we found some gene sets were identified in both males and females (Table S4). For example, the enrichment of the oligodendrocyte marker gene set was identified in both doses in both sexes; however, most of these genes were Na-negative in females but Na-positive in males, demonstrating dimorphic responses between sexes after TSA treatment (Table S4). These data suggest that even though the same gene set commonly responds to TSA treatment, individual genes have a complex, sex-specific response pattern.

**Figure 8:**
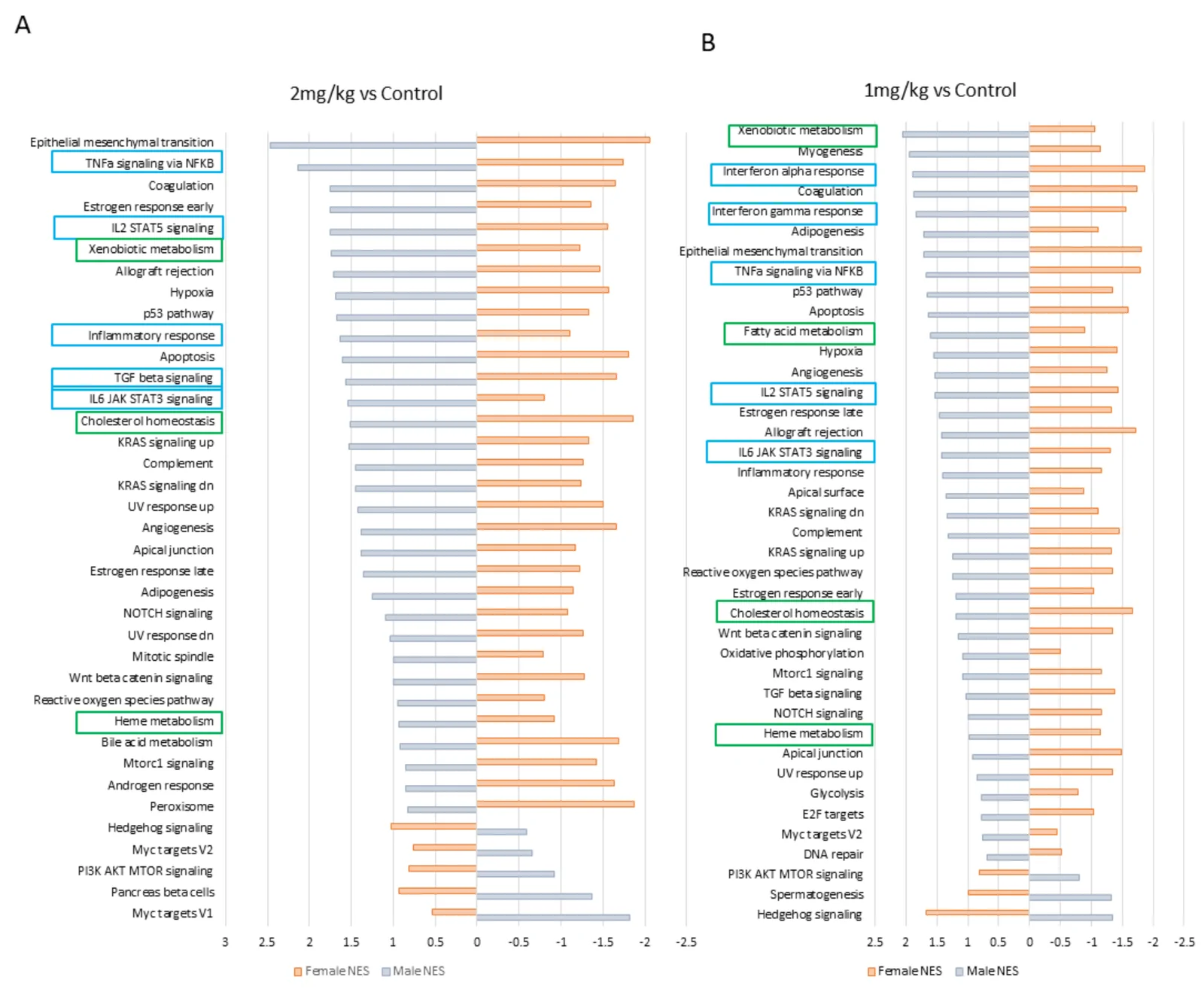
The bar graph shows only the functions that were enriched in opposite directions in male and female HAD1 rats (**A**) after 2 mg/kg treatment and (**B**) 1 mg/kg treatment with TSA. Gray indicates male normalized enrichment scores (NES), and orange indicates female NES. Important functions that are represented in both treatment groups are indicated in blue and green boxes.

Hallmark gene set analysis identified additional biological processes that respond to TSA treatment at 2 mg/kg (Figure S6) and 1 mg/kg (Figure S7) in males and females. In males administered the 2 mg/kg dose, immune-related signaling like IL6 JAK STAT3 signaling, inflammatory response, IL2 STAT5 signaling, interferon γ and α response, and TNFα signaling via NFκB was observed (indicated by blue box in Figure 8A), suggesting a strong pro-inflammatory response. In females after 2 mg/kg TSA treatment, interferon γ and α responses were similarly identified, but these signaling pathways responded differently in females and had negative normalized enrichment scores (NES) (Figure 8A). The data indicate a sex difference in terms of inflammatory response, with females having less pro-inflammatory response. For the 1 mg/kg dose, metabolism-related functions are significantly affected, with pathways such as heme metabolism, oxidative phosphorylation, cholesterol homeostasis, fatty acid metabolism, adipogenesis, and xenobiotic metabolism altered in both males and females but with different activation directions (indicated by green box, Figure 8B). More details of additional pathways between males and females are shown in Figures S6 and S7.

GSEA analysis identified several hallmark genes and microRNAs with positive or negative enrichment scores, and gene expression FC was incorporated to facilitate interpretation. We further performed STRING analysis on these genes to identify enriched protein–protein interaction (PPI) networks and evaluate their functional coherence. In general, larger input gene sets allow for better-resolved PPI network enrichment. The following two categories correspond to treatment groups associated with reduced ethanol intake.

**Figure 9:**
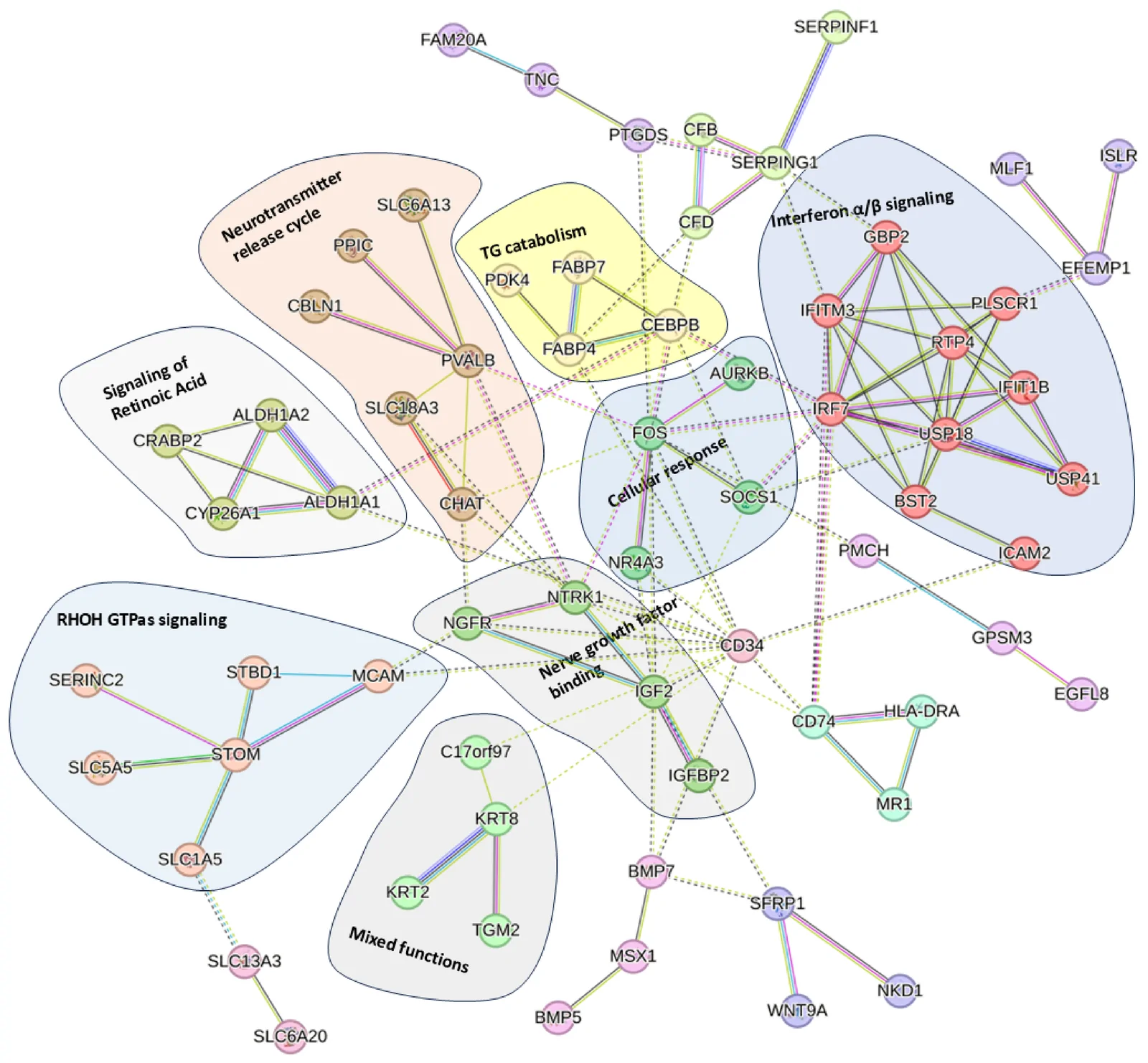
STRING analysis predicts the protein–protein interaction (PPI) network using hallmark genes (N = 99) with a Na-positive cutoff at 0.4 after 2 mg/kg TSA in males. Identified interconnected clusters and cluster members with no connection with other members were excluded. Genes involved in the same function are color-coded with the same hue and circled. Solid lines indicate direct physical interaction, and dashed lines indicate two molecules affecting each other through one or more intermediate or regulatory steps.

Using hallmark genes (N = 99) with a Na-positive cutoff of 0.4 following 2 mg/kg TSA treatment in males, the PPI network revealed preferential induction of several functional clusters, each enriched with multiple genes (Figure 9). Enrichment of interferon signaling (IRF7, ICAM2, USP18) suggests attenuation of ethanol-induced dysregulated neuroimmune responses through activation of IRF7 and induction of USP18, thereby protecting synaptic integrity. This cluster links CD34 with IGF2 signaling, which promotes memory extinction and synaptic stabilization. In addition, WNT/BMP signaling (including BMP5 and BMP7) contributes to structural plasticity and dendritic remodeling. The FOS-centered module (including NR4A3) and growth factor pathways (NTRK1, NGFR) suggest epigenetic priming of transcriptional responsiveness rather than acute activation alone. Metabolic genes (PDK4, FABP4/7) and ALDH1A1/A2 indicate reprogramming of ethanol-induced metabolic dysfunction, improving glucose and lipid utilization as well as redox balance. Collectively, these pathways likely represent a compensatory or normalization program that restores synaptic, immune, and metabolic homeostasis disrupted by ethanol exposure (Figure 9).

**Figure 10:**
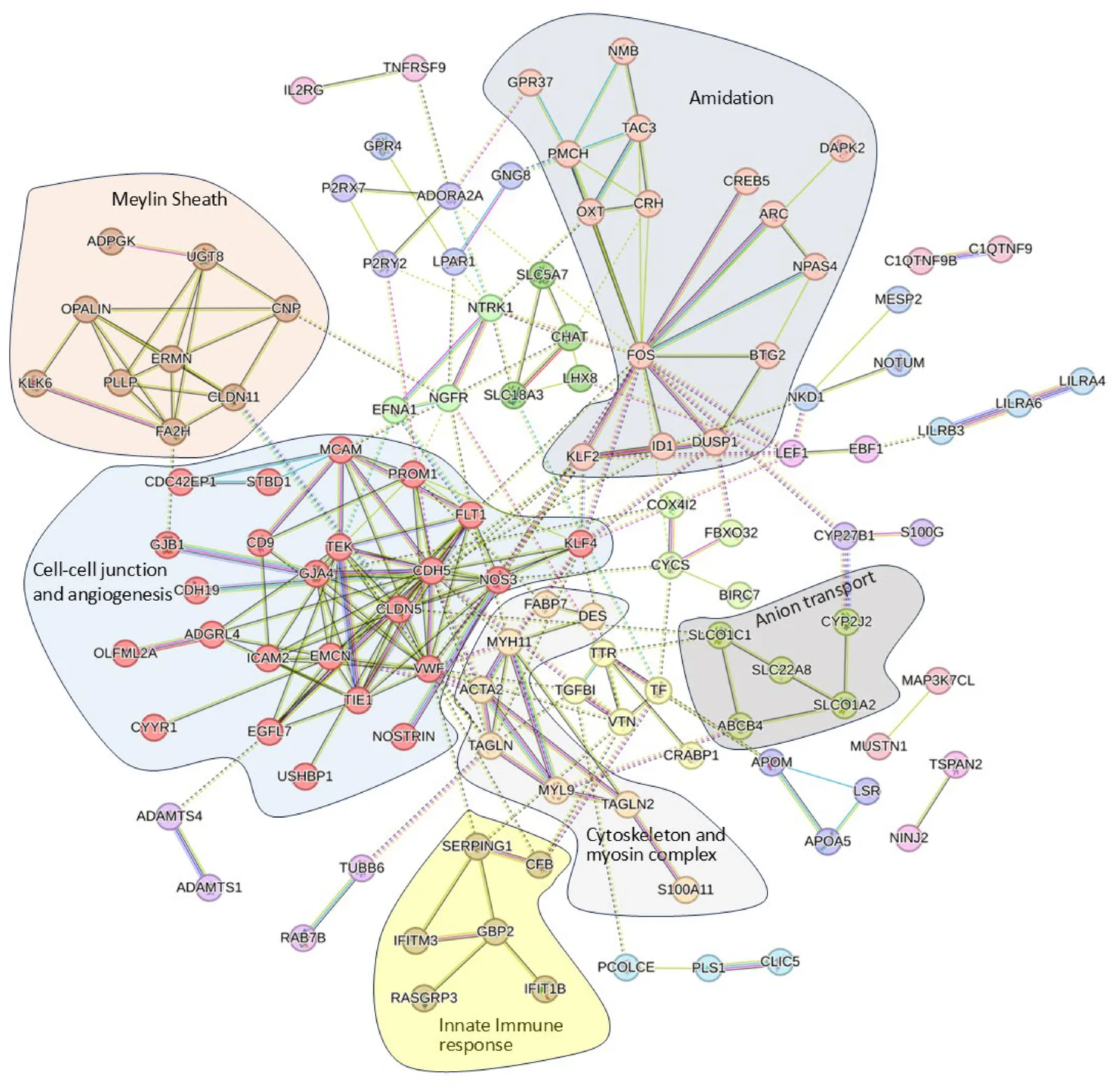
STRING analysis predicts the protein–protein interaction (PPI) network using hallmark genes (N = 155) with a Na-negative cutoff at 0.4 after 2 mg/kg TSA in females. Identified interconnected clusters and cluster members with no connection with other members were excluded. Genes involved in the same function are color-coded with the same hue and circled. Solid lines indicate direct physical interaction, and dashed lines indicate two molecules affecting each other through one or more intermediate or regulatory steps.

In contrast, in females treated with 1 mg/kg TSA, genes with a Na-negative cutoff of 0.4 (N = 155) revealed multiple functionally connected gene sets that were significantly enriched among downregulated genes, indicating coordinated suppression at the pathway level (Figure 10). The network was enriched for pathways related to cell–cell junctions and angiogenesis, suggesting remodeling of vascular, endothelial, and angiogenic signaling. The network was centered on transcriptional regulators such as FOS, ARC, and CREB5, indicating downregulation of immediate early gene expression. Suppression of stress-related neuropeptides (CRH, OXT, PMCH, etc.) may reduce stress signaling and motivational drive for ethanol consumption (Figure 10).

Collectively, these results demonstrate that 2 mg/kg TSA treatment in males induces adaptive transcriptional reprogramming, whereas 1 mg/kg TSA in females acts as a functional “brake,” leading to reduced ethanol intake over 24 h in males and reduced 4 h intake in females.

## 4. Discussion

Recently, our co-authors have investigated the TSA dose response in chronic ethanol drinking models; the overall findings are that 1 mg/kg TSA/day more significantly reduced drinking in females in 4 h ethanol consumption; and 2 mg/kg TSA/day significantly decreased drinking in males, measured by 24 h ethanol consumption [43]. To understand molecular alterations, our current RNA-seq profiling was carried out using the NAcSh of the same rats from the previous experiment. Samples were only from the 1 mg/kg and 2 mg/kg TSA doses, as those were the effective doses. Similarly to a recent study, a reduction in ethanol intake was seen with these two doses of another epi-modification drug. [42]. We found that the effect of TSA treatment on gene expression alteration is dose-specific and sex-specific. Identified pathways show some similarities between doses and sex, such as the inflammatory response network, but more divergences between sexes exist. Pathways centered by *Hdac* and *Fkbp5* were significant in males, while estrogen- and insulin-centered pathways were significant in females. Gene expression analysis provided some explanations and potential mechanisms of sex specificity and dose sensitivity. One surprising finding from both DEG analysis and GSEA indicates that some of the same genes or gene sets could respond in opposite directions between males and females after TSA treatment.

### 4.1. TSA Treatment Significantly Altered the Expression of Genes with Functions Relevant to Ethanol Consumption

Previously, multiple epigenetic mechanisms in the regulation of ethanol consumption were proposed including modification of cholinergic interneurons, adjustment of neurokinin-1 receptor sensitivity to stress, and modification by HDAC2 [31,74,75,76]. In our current study, the behavioral effects of TSA on ethanol consumption were accompanied by substantial transcriptomic remodeling in the NAcSh, revealing sex- and dose-specific gene networks associated with neuronal adaptation, neuroimmune signaling, and myelin regulation. This provides new mechanistic insights into how HDAC inhibition may attenuate AUD-related behaviors.

According to our and others’ publications, transcriptome profiling using brain tissues tends to yield DEGs with smaller FCs, e.g., Log_2_ FC 0.1–0.3 [77,78,79]. In this study, we found some top candidate genes with Log_2_ FC 2–5, suggesting they are primary targets for further investigation in epigenetic regulation of ethanol consumption. Notably, genes with high FC could drive ethanol consumption. In males, we observed significantly increased (*Oxt*, *Ntrk1*) and decreased (*Ttr*, *Fst*) expression after 1 mg/kg TSA treatment and increased (*Serpinf1*, *Ifi27l2b*) and decreased (*Alkbh8*, *Mc3r*) expression after 2 mg/kg TSA treatment. In females, significantly increased (*Tnfsf15*, *Sult1a1*) or decreased (*Pmch*, *Apold1*) expression was observed after 1 mg/kg TSA treatment, and increased (*Tmem179*, *Bdnf*) or decreased (*Hbb*, *Acta2*) expression was observed after 2 mg/kg. Some of these genes are noteworthy, as they play roles in ethanol consumption, including *Oxt*, *Pmch*, *Ttr*, and miR433 [80,81].

OXT affects ethanol-related tolerance, withdrawal, craving, and anxiety and is a candidate for AUD treatment [82,83,84,85]. Recent research has furthered our understanding of the mechanism and shown that releasing OXT in VTA reduces ethanol consumption [86]. In our study, *Oxt* increases by almost 4-fold (log_2_ ratio is 1.98) in males but decreases by a similar FC in females when comparing 1 mg/kg TSA to the control (Figure 1 and Figure 2). Concordantly, the sex difference was also observed in previous research when studying *Oxtr* knockout mice, response to OXT administration, and OXT blood concentration in humans [87,88,89]. Previously, our own study also found downregulation of *Oxt* in the NAc of females, but not males, of a congenic animal model [90]. One explanation could be due to the interplay between oxytocin and sex hormones, as estrogen receptor translocation to the nucleus regulates *Oxt* gene expression [91,92]. Epigenetic modifications of *Oxt* were found in stress responses, including DNA methylation of oxytocin, epistatic interaction between oxytocin and dopamine-related genes in humans, and alleviation of cognitive impairment in drug-induced disease animal models [93,94,95,96]. We interpret that Oxt expression through TSA treatment plays an important role in regulating ethanol consumption.

In the NAcSh of females after 1 mg/kg treatment, *Pmch*, *Oxt*, *Npas4*, and *Fos* decreased from 1.24× to 2.28× log_2_ FC (Figure 2A). *Pmch* encodes melanin-concentrating hormone (MCH), a hypothalamic neuropeptide projecting strongly to NAcSh, VTA, and limbic circuits. MCH projections heighten hedonic value of food/ethanol through NAc pathways [97]. MCHR1 enhances ethanol reward; blocking MCHR1 reduces ethanol preference and dopamine-linked signaling [98,99]. Ethanol and other drugs upregulate MCH systems, creating a reinforcing loop [100]. Additionally, MCH influences energy and reward responses, and MCH-driven motivated behaviors differ by sex [101]. Thus, downregulation of *Pmch* after 1 mg/kg TSA treatment in females likely diminishes the effect of ethanol on incentive salience and aligns with reduced ethanol consumption. In addition to the above discussion of Oxt, downregulation of Oxt may reduce ethanol–dopamine coupling and indicate successful disengagement of ethanol reinforcement circuits. Neuronal PAS Domain Protein 4 (*Npas4*) is an activity-dependent transcription factor that regulates excitatory–inhibitory balance, synaptic remodeling, and memory encoding. Downregulation in the NAc reduces conditioned reward behaviors and slows acquisition of drug reinforcement through negative regulation of the enhancer of *Npas4* gene by HDAC5 [102]. This profile closely resembles punishment-sensitive/low-compulsive drinking phenotypes [103]. These observations support a network-level reduction in ethanol reinforcement capacity and transcriptional decoupling of ethanol experience from memory formation (*Npas4* and *Fos*) aligned with reducing ethanol consumption.

TTR is synthesized predominantly by the liver, but as a major brain-derived transport protein, it is also produced by the choroid plexus of the brain. It is essential for thyroid hormone and retinol delivery to the central nervous system (CNS) and participates in the pathological process of amyloidosis [104,105,106,107,108]. Ethanol disrupts choroid plexus structure and function, and circulating levels of choroid plexus-derived proteins are decreased during inflammatory conditions and malnutrition [109,110,111]. A mouse model study found that *Ttr*, as a thyroid hormone synthesis pathway gene, was downregulated in brains of the alcohol drinking group when compared to the control group [110]. Previously, we found that TTR upregulation is associated with decreased drinking in a congenic animal model [90]. In the current study, only males given 2 mg/kg TSA treatment had significantly increased TTR (3.37×) expression; interestingly, these male rats were found to have decreased 4 h and 24 h ethanol consumption [43]. Thus, the congenic genetic model, which was derived from ethanol-preferring rats and HAD1 rats after TSA treatment, along with the above studies, suggests that altered TTR signaling is likely to contribute to neuroendocrine imbalance and cognitive dysfunction associated with chronic ethanol exposure and AUD [90,108]. We conclude that ethanol exposure indirectly reduced TTR expression and function, and 2 mg/kg TSA treatment in males restored TTR expression, at least partially explaining the decrease in drinking in these rats, possibly through epigenetic and/or neuroendocrine regulation mechanisms.

Recently, miRNA-mediated repression of stress-response genes has been identified as a critical mechanism in chronic ethanol exposure and relapse biology [112]. In the current study, we found that miR-433 is on the top hallmark gene list and significantly increased only after 2 mg/kg TSA treatment. Even though there is no direct evidence between ethanol exposure and miR-433 expression, some other studies suggest its important role as a post-transcriptional regulator that primarily controls neuronal survival, autophagy, and apoptosis under stress conditions [113,114]. Overexpression of miR-433 could rescue the Aβ-induced inhibition in neuronal viability in neuroblastoma cell lines [114]. Additionally, miR-433 is part of the miRNA cluster, a locus repeatedly shown to be epigenetically sensitive to ethanol exposure, suggesting vulnerability of miR-433 to regulation [115]. Given the established role of miR-433 in regulating apoptosis and autophagy and its increased expression after a high dose of TSA, it is reasonable to consider it functionally relevant to reduced neuronal toxicity and increased viability. Moreover, miR-761 should also be considered as another candidate for ethanol-epigenetic regulation. miR-761 is a stress-responsive, epigenetically active miRNA that suppresses *HDAC*-dependent transcriptional programs and limits pathological survival signaling. It binds the 3′UTR of *HDAC1* and was found to be downregulated in cancer, repressing its expression. We acknowledge that these observations are preliminary. Nevertheless, the identification of miR-433 and miR-761, in addition to the differentially expressed genes, represents a novel finding and highlights these miRNAs as intriguing candidate regulators that warrant further investigation as potential therapeutic targets in ethanol-related neurobiological processes.

### 4.2. TSA Treatment Altered Immune, HDAC, and Metabolism-Associated Pathways, Leading to Neurotransmitter and Neuronal Modification Underlying Reduction of Ethanol Consumption

#### 4.2.1. TSA-Induced Immune-Related Pathway Regulation Is Significant

Previous reports demonstrate that TSA possesses anti-inflammatory and neuroprotective properties in the CNS and may act through HDAC regulation and ethanol-induced neuroimmune responses. In our alcohol model, immune-related processes emerged as a shared response across both sexes and TSA treatment doses. However, the underlying immune regulatory mechanisms appeared to be sex-specific. Multiple immune-related signals are noteworthy, including IL6/JAK/STAT3 signaling, IL2/STAT5 signaling, IFNγ response, IFNα response, and TNFα signaling via NFkB; however, these signaling pathways show divergent activation patterns between males and females, characterized by negative NES values in males and positive NES values in females (Figure 8). Upregulation of IL6/JAK/STAT3 signaling results in chronic inflammation, promotes cancer, and promotes expression of anti-apoptotic and proliferative genes [116,117]. Upregulation of IL-2/STAT5 signaling promotes Treg stability, development of Th1 and Th2, and T cell survival, which are essential for immune tolerance [118]. Consistently, TSA has immunomodulatory effects including induction of immune tolerance and Treg expansion [37,119,120]. In males, both doses enhance IL-6 signaling, inflammatory response, and TNFα signaling via NFκB, all of which are responses dampened in females (Figure 8A,B). Additional pathways altered the NTRK1-HDAC-RELA network, suggesting neuroinflammation is partly through NFκB activation and could affect other gene expressions (Figure 4B). Notably, genes related to immune function were upregulated, including *Serpinf1* and *Ifi27l2b*, as well as downregulated, in the case of *Sult1a1*. *Serpinf1* plays roles in neuroprotection and anti-inflammation. TSA could activate neurotrophic pathways and upregulate interferon-stimulated genes, which may connect to better counter oxidative stress and immune modulation. Downregulation of *Sult1a1* could slow sulfation of neurotransmitters and ethanol metabolites and reduce ethanol-induced rewarding effects. Consistently, *STRING* network analysis suggests a system where neuronal signaling (cholinergic and neurotrophic) is tightly integrated with immune activation (interferon and complement pathways) and metabolic regulation (insulin, PPAR, IGF, retinoic acid, transporters) (Figure 9). Thus, sex-specific immune regulation by TSA treatment is evident.

#### 4.2.2. TSA Treatment Affects Myelin Remodeling Gene in Sex-Dependent Manner

Previous findings suggest that chronic ethanol exposure can induce myelin remodeling within reward-related brain regions, including the NAc. Recent studies have demonstrated that long-term ethanol consumption enhances myelination and oligodendrocyte maturation in the NAc, suggesting a potential role for myelin plasticity in the maintenance of ethanol-drinking behaviors [121]. The selective breeding process increased ethanol consumption in each generation, Recent findings raise the possibility that enhanced myelination may contribute to this phenotype, and it will be interesting to examine this possibility in animal models for AUDs [121,122].

In addition, recent evidence has revealed sex-dependent microglial responses to chronic ethanol exposure and stress, particularly within the hippocampus and amygdala, indicating that glial regulation may contribute to sexually dimorphic, neurobiological adaptations associated with ethanol use [123]. These findings raise the possibility that HDAC inhibition by TSA may influence ethanol-related behaviors through modulation of glial and oligodendrocyte-associated pathways. Consistent with this hypothesis, enhanced expression of myelin-related genes may contribute to the restoration of ethanol-induced oligodendrocyte dysfunction, white matter abnormalities, and impaired neuronal connectivity, thereby altering reward-circuit plasticity and ethanol consumption.

Notably, our GSEA analysis identified significant enrichment of the myelin sheath gene cluster in females treated with 1 mg/kg TSA. Further examination of individual genes revealed a striking sex-dependent pattern, whereby multiple myelin-associated genes (e.g., OPALIN, ERMN, UGT8, FA2H, CNP, CLDN11, and PLLP), including markers of mature oligodendrocytes and myelin structure, were generally downregulated in females but upregulated in males following TSA treatment (Tables S4 and S5). This pattern suggests that TSA differentially regulates oligodendrocyte- and myelin-related transcriptional programs between sexes. The observation is consistent with recent studies highlighting sex differences in glial responses and ethanol-associated myelin remodeling. Collectively, these findings suggest that HDAC inhibition may promote a pro-myelinating oligodendrocyte transcriptional program in males, characterized by increased expression of genes involved in myelin formation, maintenance, and oligodendrocyte maturation. In contrast, females exhibited an opposing transcriptional response. Although direct assessment of myelin structure and oligodendrocyte morphology was not performed in the current study, the observed sex-dependent regulation of myelin-related genes suggests that differential modulation of oligodendrocyte biology may contribute to the sexually dimorphic effects of TSA on ethanol-related phenotypes.

#### 4.2.3. HDAC Network Plays a Role in Reducing Ethanol Consumption After 2 mg/kg TSA Treatment in Males

Given that 2 mg/kg TSA is the most effective dose for reducing ethanol consumption in males measured by 24 h intake and in females by 4 h intake at multiple testing days, the data highlight the HDAC-centered pathway and the potential gene-regulatory mechanisms in the NAcSh underlying this effect (Figure 4B). Our discussion will focus on TSA’s alteration of neurotransmitters, neuron plasticity and survival, and cellular stress signaling. In this pathway, a cluster of genes includes *Ntrk1* (TrkA) and *RelA* directly connected to HDAC, and several neurotransmitter receptors (CHRM4, GRM2, MC3R) connected to ADCY and G-protein coupled receptors (GPR4 and RGS members). Increased *Ntrk1* signaling suggests enhanced activity-dependent plasticity, including accumulation of acetylcholine neurotransmitter loading, promotion of acetylcholine synthesis, and release through NGF and TrkA-dependent regulation [124,125,126]. Other genes, like increased Rgs6 and Rgs12 and decreased Rgs and Rgs17, affect G-protein signaling and dopamine/glutamate modulation. Upregulation of GRM2 activation reduces presynaptic glutamate release, dampening excitatory drive in reward circuits, and previously, we found that loss of *Grm2* is associated with high ethanol consumption in rats [127]. *Chrm4* upregulation modulates cholinergic tone, which can reduce dopamine release in mesolimbic pathways. Both neuropeptide receptors decrease the reinforcing properties of ethanol. Several factors affect stress response signaling. For example, *Ntrk1* interacts with RNA-processing proteins (*Cwf19l2*, *Gpatch4*), stabilizing stress responses. Furthermore, NTRK1 activates RelA and is linked to stress and inflammatory responses. Additionally, decreased FKBP5 increases stress adaptation (Table 2) [128,129]. Collectively, these results suggest a possible association between TSA-induced transcriptional changes in neurotransmission- and stress-related pathways and the reduced ethanol consumption observed following 2 mg/kg TSA treatment; however, a causal relationship remains to be established. An additional explanation is the promotion of neuronal differentiation and survival in males after 2 mg/kg dose through Sirtuin signaling pathway regulation. Sirtuins are a family of seven highly conserved proteins (SIRT1–SIRT7) found in mammals that play critical roles in gene expression, cellular metabolism, stress resistance, and DNA repair. They are NAD^+^-dependent enzymes, and their primary activity is deacetylation. Sirtuins, especially SIRT1 and SIRT2, have been shown to play a key role in addiction-related brain changes, particularly in the NAc. Chronic cocaine and morphine use increases expression of SIRT1 and SIRT2 in the NAc [130]. Overexpression of SIRT1 or SIRT2 in the NAc enhances drug reward, while knockdown of SIRT1 reduces drug-seeking behavior [131]. Thus, inactivation of this pathway would reduce deacetylation activity, modify the epigenetic makeup of related genes, and reduce ethanol reward.

In contrast, TTR downregulation and Fkbp5 upregulation following 1 mg/kg TSA treatment show expression patterns opposite to those observed with 2 mg/kg TSA, suggesting a potential enhancement of stress-related signaling. Furthermore, downregulation of FMR1, *Cacnb4*, *Fgf14* and *Pcdhg8* may adversely affect synaptic regulation and network stability. Together, these changes may reduce the capacity of 1 mg/kg TSA to normalize executive and inhibitory synaptic functions in the NAcSh, which could contribute to the lack of a measurable effect on ethanol consumption.

#### 4.2.4. Estrogen-Related Hormone Signaling Dampens Reward Sensitivity and Decreases Ethanol Consumption in Females in 1 mg/kg TSA Treatment

Given that 1 mg/kg TSA is the most effective dose for reducing ethanol consumption measured by 4 h intake in females, we further explored molecular evidence underlining this. In addition to previously discussed DEGs with high FC (*Pmch*, *Oxt*, *Npas4* and *Fos*), top pathways also strengthen the mechanistic understanding of the role of metabolism alteration after TSA treatment. Notably, some metabolism-related pathways were decreased, such as the cholesterol biosynthesis-related Wnt/Ca^+^ pathway (Figure 5B). Our observation is consistent with the evidence that TSA reduced cholesterol levels, increased its catabolism, and controlled SREBP-driven cholesterol biosynthetic gene expression [132,133]. We emphasize a metabolism-associated pathway showing estrogen-centered signaling nodes interacting with metabolic and neuroendocrine regulators (Figure 5C). Genes in this pathway were grouped into hormonal, metabolic, stress, and reward clusters based on canonical biological function and network localization. The hormonal cluster consists of ESR (estrogen receptor) and insulin for glucose control. Previously, TSA has been shown to enhance estrogen receptor-dependent transcription by increasing chromatin accessibility, restoring ER expression, and stabilizing acetylated ER complexes [134,135]. Another study revealed gene expression differences in NAc between sexes, particularly ESR upregulated fivefold only in females [48]. Decreased SLC16A1 (−1.18×) for insulin/estrogen-responsive metabolite transport and transferrin receptor (TFRC, −1.15×) for hormone-regulated metabolic signaling and cellular iron uptake both support metabolic signal alteration. Furthermore, the metabolic cluster includes genes that regulate metabolism (increased ACSL3 for fatty acid metabolism), transporters (decreased SLC22A8 and SLC38A2), and decreased ATPase for energy utilization, further interpreted as metabolism-associated changes. This pathway also includes cellular stress response hub genes (e.g., WDR19, VCPIP1), along with reward/cytoskeleton and neuro-mobility genes (CAV2, OCLN). In summary, 1 mg/kg TSA may decrease ethanol consumption by epigenetically enhancing estrogen-driven hormone and metabolic networks in a short period of time, leading to reduced stress responsiveness and altered reward processing.

#### 4.2.5. GR, Fkbp5 and BDNF Epigenetic Modification After TSA

Previous work suggests that epigenetic mechanisms (HDACs, DNMTs, histone acetylation) are sexually differentiated and interact with steroid hormones to shape circuit plasticity [32,33,136]. In the current study, glucocorticoid (GC) and its receptor GR are a top causal network affected by the 2 mg/kg dose of TSA in males, and estrogen is one common upstream regulator in all doses and sexes. We predict that glucocorticoid and estrogen actions occur synergistically and mediate TSA response. Aside from the previously mentioned HDAC pathway, an additional significant network is the Fkbp5, Hsp90, Hsp70, and co-factors of GR centered pathway (Figure S4B). The FKBP5 protein is a cochaperone of HSP90 and regulates stress response along with other HPA genes such as Nr3C1, Hsp90, and Crh [137,138]. Previously, FKBP5 was found to affect neurite outgrowth, neuron morphology, and hippocampus formation [139,140,141]. GC and GR are involved in mediating ethanol-seeking, compulsive drinking, and abstinence, and we have found a sex-specific difference in response to ethanol exposure in *Fkbp5* KO mice [68,142,143]. *Fkbp5* is directly and indirectly connected to other gene clusters that result in functional alterations. Increased Fkbp5, *Prkab2*, and other genes are associated with neuronal development. Other affected functions include RNA binding and process, synaptic transmission and cytoskeletal-related proteins and immune-related genes. We found that a 2 mg/kg TSA dose decreases *Fkbp5* in the NAcSh of males and increases it in females; however, a 1 mg/kg dose induces similar increases in both sexes, which could explain sex differences after TSA treatment in Fkbp5-associated functions like stress adaptation.

Previous research has linked increased intracellular FKPB5 protein expression and faster antidepressant response in humans with specific genotypes, as well as an increase in mRNA in specific brain regions after stress in mouse models [144,145]. The interplay between stress and ethanol use results in epigenetic modification of gene expression in reward circuits, neurobehavioral deficits, and disease progression [146,147,148]. FKBP5 has been found to indirectly participate in stress-mediated epigenetic programming through the HPA axis [149,150] and through its action on GR [137,151,152]. Directly, *FKBP5* epigenetic modification is associated with stress resiliency, cardiovascular disease, and post-traumatic stress disorder [150,153,154,155]. In particular, FKBP51 controls phosphorylation and thus the activity of DNMT1, suggesting it directly contributes to epigenetic programming [156].

Further, we found evidence that the BDNF network is implicated in the TSA response and that BDNF is an upstream regulator, which may affect gene expression of identified DEGs (Figure S4A). Our study found that a 2 mg/kg dose of TSA increased *Bdnf* and *Irf7* expression in both sexes, which may enhance neuron and neurite growth and immune regulation. Various studies have supported histone deacetylation of BDNF in synaptic plasticity [157]. Consistently, TSA treatment was found to increase BDNF in a high fat diet-fed model [132]. Additionally, the BDNF pathway showed that several nuclear receptor family member expressions were altered. Lower expression of nuclear receptor subfamily 4 group A member 1 (*Nr4a1*), KLF transcription factor 10 (a transcriptional repressor and effector of TGFβ signaling), and cAMP responsive element binding protein (Creb) was observed (Figure S4A). Creb has histone acetyltransferase binding activity and is involved in AUD, obsessive-compulsive disorder, and drug dependence. Interestingly, FKBP5 expression is inversely correlated with both global and BDNF methylation, and stress-induced FKBP5 protein levels result in increased BDNF expression [156]. Thus, this study suggests that TSA treatment modification of BDNF, FKBP5, and related pathways may be one of the potential mechanisms for reducing ethanol consumption in animal models. Thus, our result is consistent with corticostriatal- and NAc-specific molecular plasticity differences between males and females; females show broader transcriptional remodeling, but males show restricted, stress-biased gene networks [158].

TSA treatment elicited dose- and sex-dependent transcriptional responses in the NAcSh that were associated with alterations in neuronal and synaptic function. The identified DEGs, gene sets, and pathways provide insights into the molecular basis of these differential responses and may help guide future investigations of sex-specific therapeutic approaches.

## Figures and Tables

**Table 1:** Top 20 Most Significant Dose Response Genes in Males and Females.

Top 20 Most Significant Dose Response Genes in Males					
Symbol	TSA 1 mg/kg	TSA 2 mg/kg	FC	*p*-Value	Gene Full Name
Positive correlation with increasing dose					
Ttr	49.3	8171.0	165.83	0.004	transthyretin
Bmp5	66.1	154.1	2.33	0.001	bone morphogenetic protein 5
Bmp7	27.3	60.4	2.21	0.009	bone morphogenetic protein 7
Col8a1	22.0	48.0	2.18	0.000	collagen type VIII alpha 1 chain
Neurod2	98.6	166.7	1.69	0.002	neuronal differentiation 2
Bmp3	101.7	169.1	1.66	0.001	bone morphogenetic protein 3
Nr4a3	245.6	392.7	1.60	0.001	nuclear receptor subfamily 4, group A, member 3
Tbr1	103.2	160.5	1.56	0.003	T-box, brain, 1
Itgbl1	94.0	145.7	1.55	0.003	integrin subunit beta like 1
Mmp14	75.5	116.4	1.54	0.006	matrix metallopeptidase 14
Negative correlation with increasing dose					
Sult1a1	641.5	347.3	−1.85	0.000	sulfotransferase family 1A member 1
Fmo2	42.7	27.6	−1.55	0.004	flavin containing monooxygenase 2
Zbtb16	431.9	284.4	−1.52	0.003	zinc finger and BTB domain containing 16
Gbx1	37.8	25.3	−1.49	0.009	gastrulation brain homeobox 1
Hmgcs2	565.0	384.0	−1.47	0.000	3-hydroxy-3-methylglutaryl-CoA synthase 2
Hif3a	68.2	48.1	−1.42	0.006	hypoxia inducible factor 3 subunit alpha
Gpatch4	779.5	575.5	−1.35	0.005	G patch domain containing 4
Fkbp5	2216.8	1673.7	−1.32	0.000	FK506 binding protein 5
Mfsd2a	597.3	459.0	−1.30	0.000	major facilitator superfamily domain containing 2A
Pex11a	651.3	501.8	−1.30	0.000	peroxisomal biogenesis factor 11 alpha
**Top 20 Most Significant Dose Response Genes in Females**					
Positive correlation with increasing dose					
Ifit1	41.0	73.1	1.78	0.005	interferon-induced protein with tetratricopeptide repeats 1
Npas4	31.6	55.8	1.77	0.003	neuronal PAS domain protein 4
Evi2b	35.8	50.6	1.41	0.006	ecotropic viral integration site 2B
Akr1cl	57.9	81.0	1.40	0.012	aldo-keto reductase family 1, member C-like
Lims2	71.6	94.1	1.32	0.011	LIM zinc finger domain containing 2
Dock6	127.1	165.5	1.30	0.004	dedicator of cytokinesis 6
Tp53i3	59.6	76.4	1.28	0.015	tumor protein p53 inducible protein 3
Fos	136.2	173.1	1.27	0.007	Fos proto-oncogene, AP-1 transcription factor subunit
Dll1	95.4	118.0	1.24	0.013	delta like canonical Notch ligand 1
P2rx7	96.7	118.5	1.22	0.014	purinergic receptor P2X 7
LOC100910973	193.4	230.4	1.19	0.006	uncharacterized LOC100910973
Egfl7	203.8	241.9	1.19	0.008	EGF-like-domain, multiple 7
Negative correlation with increasing dose					
Styx	26.5	17.9	−1.48	0.010	serine/threonine/tyrosine interacting protein
Fam134b	84.0	63.8	−1.32	0.006	Fam134b
Marcksl1	211.5	167.5	−1.26	0.010	MARCKS-like 1
Ier5l	283.5	232.6	−1.22	0.005	immediate early response 5-like
Sox11	159.1	130.9	−1.22	0.012	SRY box 11
Stx7	396.7	336.9	−1.18	0.007	syntaxin 7
Fam181b	645.5	554.6	−1.16	0.002	family with sequence similarity 181, member B
Hopx	561.4	484.8	−1.16	0.011	HOP homeobox

**Table 2:** Expression of DEGs in Common Between Both Doses and Sexes.

Gene Symbol	Male Control	Male 2 mg/kg	Female Control	Female 2 mg/kg
*Irf7*	2.83	4.02	2.75	3.78
*Bdnf*	1.30	1.72	1.27	1.73
*Dbp*	131.59	156.05	148.17	128.29
*RGD1559896*	85.22	101.75	98.36	84.08
*Opalin*	63.65	83.48	84.27	64.55
*Pltp*	39.93	45.04	46.40	39.90
*Nr1d1*	33.21	39.28	42.83	36.29
*Fam129b*	18.32	21.19	19.92	17.58
*Mcam*	13.71	18.41	17.94	13.94
*Arc*	11.33	14.58	16.44	12.48
*Dusp1*	11.56	14.00	18.41	13.78
*Nkd1*	8.94	12.33	11.12	8.93
*Pdpn*	9.82	11.91	10.69	8.47
*Klf10*	7.00	8.89	8.30	6.98
*Scd1*	7.06	8.25	8.82	7.49
*Fgfrl1*	6.16	7.29	7.18	5.09
*Fos*	4.38	6.65	8.97	6.15
*Rab7b*	3.30	4.24	4.00	3.09
*Stbd1*	1.19	1.67	1.39	0.93
*Mob3b*	0.96	1.25	1.19	0.92
*Gjb6*	41.11	33.87	30.85	39.87
*Fkbp5*	33.24	28.46	23.11	32.54
*Gpatch4*	21.60	18.16	15.50	19.85
*Sult1a1*	25.16	16.86	16.61	25.63
*Pex11a*	15.15	13.14	11.89	14.17
*Mfsd2a*	15.19	12.65	11.74	15.62
*Lonrf3*	7.09	5.93	5.15	6.72
*Fst*	1.56	1.04	0.87	1.37
*Plce1*	1.05	0.88	1.00	1.24
**Gene Symbol**	**Male Control**	**Male 1 mg/kg**	**Female Control**	**Female 1 mg/kg**
*Fkbp5*	33.24	37.54	23.11	32.43
*Nxn*	19.8	24.48	16.68	18.48
*Acss3*	0.76	1.17	0.91	1.05
*Adamts4*	8.8	11.14	11.08	8.19
*Atf5*	46.83	57.34	47.82	41.34
*Bspry*	13.81	17.22	15.02	12.27
*Bst2*	4.58	6.85	5.59	4.09
*Cd82*	35.54	45.08	38.58	32.33
*Cdc42ep1*	17.19	20.82	19.97	15.08
*Csrp1*	322.11	403.52	317.95	268.06
*Dusp1*	11.56	14.17	18.41	14.19
*Elovl1*	38.48	47.3	42.77	34.62
*Fhdc1*	2.81	3.38	3.5	2.89
*Gamt*	41.94	51.91	40.37	35.74
*Gltp*	39.37	51.17	42.61	34.44
*Josd2*	51.3	66.46	50.63	41.84
*Map4*	84.12	96.99	91.71	81.22
*Oxt*	0.81	2.84	5.18	1.14
*Rassf3*	2.86	3.59	3.45	2.67
*Tagln*	16.36	22.95	18.18	13.25
*Tagln2*	14.34	19.52	15.24	11.54
*Vwa1*	15.37	17.27	15.62	14
*Wipi1*	19.22	22.3	22.04	18.94
*Pqlc3*	1.69	2.55	2.36	1.77
*Ninj2*	10.66	14.1	11.89	8.32
*Ifitm3*	6.93	11.33	10.57	6.15
*Adpgk*	34.19	38.11	32.96	24.09
*Map9*	25.58	21.93	26.02	29.47
*Dpp10*	47.35	39.48	49.28	53.87
*Fam49a*	44.62	38.5	44.54	53.75
*Fam65b*	23.53	19.75	22.93	25.73
*Napb*	140.41	119.72	139.44	159.48
*Rasgrf2*	8.71	7.05	9.75	12.69
*Slc4a10*	47.36	40.9	49.4	54.71
*Sphkap*	15.98	12.74	16.36	19.47
*Stxbp6*	21.49	17.5	21.09	24.13
*Rnf138*	6.38	5.36	6.4	5.64
*Plxnd1*	4.33	3.73	4.97	4.2

Red indicates increased, and blue decreased, gene expression after TSA treatment compared to control.

**Table 3:** Common IPA Pathways by Dose in Males and Females.

	Common Pathways Between Doses	−Log *p*-Value	
	Ingenuity Canonical Pathways	2 mg/kg	1 mg/kg
Male	Embryonic Stem Cell Pluripotency	4.27	1.5
	Axonal Guidance Signaling	3.74	1.6
	Acute Phase Response Signaling	2.69	1.48
	Aryl Hydrocarbon Receptor Signaling	2.65	1.27
	LXR/RXR Activation	2.37	1.49
	IL-6 Signaling	2.29	2.8
	Neuropathic Pain Signaling In Dorsal Horn Neurons	5.34	3.24
Female	Corticotropin Releasing Hormone Signaling	5.23	2.63
	Regulation of IL-2 Expression in Activated and Anergic T Lymphocytes	3.47	3.57
	Hepatic Fibrosis Signaling Pathway	3.00	4.00
	Protein Kinase A Signaling	2.83	3.65
	ILK Signaling	2.76	2.73
	Cellular Effects of Sildenafil (Viagra)	2.73	3.56
	PI3K Signaling in B Lymphocytes	2.63	5.31
	ERK/MAPK Signaling	2.62	2.51
	Tight Junction Signaling	2.27	6.3
	Cardiac Hypertrophy Signaling (Enhanced)	2.21	3.47
	Wnt/Ca^+^ Pathway	2.08	5.76
	Regulation Of The Epithelial Mesenchymal Transition By Growth Factors Pathway	2.07	3.27
	Synaptic Long Term Depression	2.06	3.25

## Data Availability

The original contributions presented in this study are included in the article/Supplementary Materials. Further inquiries can be directed to the corresponding author.

## Supplementary Materials

The following supporting information can be downloaded at: https://www.mdpi.com/article/10.3390/cells15161494/s1, Supplemental Figure S1. Schematic diagram of testing procedure. Subjects were 60 days old at the start of the experiments, and the rats were allowed to consume 15% and 30% ethanol concurrently with water for 8 weeks of 24 h continuous access drinking. All rats received free access to standard laboratory chow and water throughout the experiments. Body weight (BW) and food intake (FI) were noted. The animals were maintained on a 12/12 h reverse dark/light cycle (lights off at 1100 h). The rats were randomly assigned to control and dose groups (1 mg/kg and 2 mg/kg) during test week, and the last week 9 of ethanol drinking was used as a baseline. Brains were snap-frozen, followed by slicing and micro-punch on slides. Supplemental Figure S2. IPA analysis identified prominent gene networks in male HAD1 rats after different doses of TSA compared to the control. (A) Immune function-related gene network connected to NF-κB complex, IRF7 and neurotransmitter after 2 mg/kg dose of TSA. (B) Network involving FKBP5, TTR, and UBR1 is prominent in male rats administered a 1 mg/kg dose of TSA. Solid lines indicate direct physical interaction, and dashed lines indicate two molecules affecting each other through one or more intermediate or regulatory steps. Supplemental Figure S3. Pathway alterations and genes differently impacted between 2 mg/kg and 1 mg/kg doses of TSA (A) in male and (B) in female HAD1 rats. Solid lines indicate direct physical interaction, and dashed lines indicate two molecules affecting each other through one or more intermediate or regulatory steps. Supplemental Figure S4. Prominent gene networks in female HAD1 rats after different doses of TSA compared to the control. (A) A gene network connecting BDNF to genes like Creb, NR4A1, and DUSP1 is significant in female rats administered a 2 mg/kg dose of TSA. (B) Network involving synaptic transmission, neuronal development and RNA process is prominent in female rats administered a 1 mg/kg dose of TSA. Green nodes indicate downregulated genes, and pink nodes indicate upregulated genes. Solid lines indicate direct physical interaction, and dashed lines indicate two molecules affecting each other through one or more intermediate or regulatory steps. Supplemental Figure S5. Overview of GSEA-enriched gene sets; each dot represents a gene set, and yellow highlighted areas are significant gene sets. (A) Male HAD1 rats after 2 mg/kg dose TSA and (B) Male HAD1 rats after 1 mg/kg dose TSA. (C) Female HAD1 rats after 2 mg/kg dose TSA and (D) Female HAD1 rats after 1 mg/kg dose TSA. A positive normalized enrichment score (NES) indicates gene set enrichment at the top of the ranked list; a negative ES indicates gene set enrichment at the bottom of the ranked list. Supplemental Figure S6. Hallmark gene set analysis identified processes that are differentially enriched after a 2 mg/kg dose of TSA in (A) males and (B) females. Supplemental Figure S7. Hallmark gene set analysis identified processes and functions that are differentially enriched after a 1 mg/kg dose of TSA in (A) males and (B) females. Supplemental Table S1. The number of identified differentially expressed genes in males and females. Supplemental Table S2. Number of gene sets identified by GSEA. Supplemental Table S3. Comparison of enrichment score of genes in oligodendrocyte markers gene set. Supplemental Table S4. Hallmark Gene Sets. Supplemental Table S5. Genes enriched in myelin sheath cluster.

## Abbreviations

The following abbreviations are used in this manuscript:

ASB13	Ankyrin Repeat and SOCS Box Containing 13
COQ10B	Coenzyme Q10B
DES	Desmin
EFHD1	EF-Hand Domain Family Member D1
HNRNPR	Heterogeneous Nuclear Ribonucleoprotein R
KANK2	KN Motif and Ankyrin Repeat Domains 2
KLF4	KLF Transcription Factor 4
NR4A1	Nuclear Receptor Subfamily 4 Group A Member 1
NR3C2	Nuclear Receptor Subfamily 3 Group C Member 2
PARN	Poly(A)-Specific Ribonuclease
PMCH	Pro-Melanin Concentrating Hormone
PRKAB2	Protein Kinase AMP-Activated Non-Catalytic Subunit Beta 2
PRR5L	Proline Rich 5 Like
RC3H2	Ring Finger And CCCH-Type Domains 2
SNTB1	Syntrophin Beta 1
STXBP5L	Syntaxin Binding Protein 5L
SPRED1	Sprouty Related EVH1 Domain Containing 1
SS18L1	SS18L1 Subunit of BAF Chromatin Remodeling Complex
LMO2	LIM Domain Only 2
TLE3	TLE Family Member 3, Transcriptional Corepressor
TTR	Transthyretin
USHBP1	USH1 Protein Network Component Harmonin Binding Protein 1
ZMYND19	Zinc Finger MYND-Type Containing 19

## References

[1] Kessler R.C., McGonagle K.A., Zhao S., Nelson C.B., Hughes M., Eshleman S., Wittchen H.U., Kendler K.S. (1994). Lifetime and 12-month prevalence of DSM-III-R psychiatric disorders in the United States. Results from the National Comorbidity Survey. Arch. Gen. Psychiatry.

[2] Cornelius J.R., Bukstein O., Salloum I., Clark D. (2003). Alcohol and psychiatric comorbidity. Recent Developments in Alcoholism.

[3] Clay J.M., Parker M.O. (2020). Alcohol use and misuse during the COVID-19 pandemic: A potential public health crisis?. Lancet Public Health.

[4] Sacks J.J., Gonzales K.R., Bouchery E.E., Tomedi L.E., Brewer R.D. (2015). 2010 National and State Costs of Excessive Alcohol Consumption. Am. J. Prev. Med..

[5] Enoch M.A., Goldman D. (2001). The genetics of alcoholism and alcohol abuse. Curr. Psychiatry Rep..

[6] Prescott C.A., Kendler K.S. (1999). Genetic and environmental contributions to alcohol abuse and dependence in a population-based sample of male twins. Am. J. Psychiatry.

[7] Hart A.B., Kranzler H.R. (2015). Alcohol Dependence Genetics: Lessons Learned From Genome-Wide Association Studies (GWAS) and Post-GWAS Analyses. Alcohol Clin. Exp. Res..

[8] Reilly M.T., Noronha A., Goldman D., Koob G.F. (2017). Genetic studies of alcohol dependence in the context of the addiction cycle. Neuropharmacology.

[9] Liu C., Marioni R.E., Hedman A.K., Pfeiffer L., Tsai P.C., Reynolds L.M., Just A.C., Duan Q., Boer C.G., Tanaka T. (2018). A DNA methylation biomarker of alcohol consumption. Mol. Psychiatry.

[10] Longley M.J., Lee J., Jung J., Lohoff F.W. (2021). Epigenetics of alcohol use disorder-A review of recent advances in DNA methylation profiling. Addict. Biol..

[11] Zhou H., Gelernter J. (2024). Human genetics and epigenetics of alcohol use disorder. J. Clin. Investig..

[12] Berkel T.D., Pandey S.C. (2017). Emerging Role of Epigenetic Mechanisms in Alcohol Addiction. Alcohol Clin. Exp. Res..

[13] Palmisano M., Pandey S.C. (2017). Epigenetic mechanisms of alcoholism and stress-related disorders. Alcohol.

[14] Moonat S., Pandey S.C. (2012). Stress, epigenetics, and alcoholism. Alcohol Res..

[15] Koob G.F., Mason B.J. (2016). Existing and Future Drugs for the Treatment of the Dark Side of Addiction. Annu. Rev. Pharmacol. Toxicol..

[16] Gatta E., Auta J., Gavin D.P., Bhaumik D.K., Grayson D.R., Pandey S.C., Guidotti A. (2017). Emerging Role of One-Carbon Metabolism and DNA Methylation Enrichment on delta-Containing GABAA Receptor Expression in the Cerebellum of Subjects with Alcohol Use Disorders (AUD). Int. J. Neuropsychopharmacol..

[17] Wang Z., Zang C., Rosenfeld J.A., Schones D.E., Barski A., Cuddapah S., Cui K., Roh T.-Y., Peng W., Zhang M.Q. (2008). Combinatorial patterns of histone acetylations and methylations in the human genome. Nat. Genet..

[18] Pandey S.C., Bohnsack J.P. (2020). Alcohol Makes Its Epigenetic Marks. Cell Metab..

[19] Bohnsack J.P., Hughes B.A., O’Buckley T.K., Edokpolor K., Besheer J., Morrow A.L. (2018). Histone deacetylases mediate GABAA receptor expression, physiology, and behavioral maladaptations in rat models of alcohol dependence. Neuropsychopharmacology.

[20] Petry N.M., Kirby K.N., Kranzler H.R. (2002). Effects of gender and family history of alcohol dependence on a behavioral task of impulsivity in healthy subjects. J. Stud. Alcohol.

[21] Zillich L., Frank J., Streit F., Friske M.M., Foo J.C., Sirignano L., Heilmann-Heimbach S., Dukal H., Degenhardt F., Hoffmann P. (2022). Epigenome-wide association study of alcohol use disorder in five brain regions. Neuropsychopharmacology.

[22] Bohnsack J.P., Pandey S.C. (2021). Histone modifications, DNA methylation, and the epigenetic code of alcohol use disorder. Int. Rev. Neurobiol..

[23] Bonsch D., Lenz B., Kornhuber J., Bleich S. (2005). DNA hypermethylation of the alpha synuclein promoter in patients with alcoholism. NeuroReport.

[24] Dulman R.S., Wandling G.M., Pandey S.C. (2020). Epigenetic mechanisms underlying pathobiology of alcohol use disorder. Curr. Pathobiol. Rep..

[25] Rodriguez F.D. (2021). Targeting Epigenetic Mechanisms to Treat Alcohol Use Disorders (AUD). Curr. Pharm. Des..

[26] Gatta E., Grayson D.R., Auta J., Saudagar V., Dong E., Chen Y., Krishnan H.R., Drnevich J., Pandey S.C., Guidotti A. (2019). Genome-wide methylation in alcohol use disorder subjects: Implications for an epigenetic regulation of the cortico-limbic glucocorticoid receptors (NR3C1). Mol. Psychiatry.

[27] Li D., Zhang Y., Zhang Y., Wang Q., Miao Q., Xu Y., Soares J.C., Zhang X., Zhang R. (2019). Correlation between the epigenetic modification of histone H3K9 acetylation of NR2B gene promoter in rat hippocampus and ethanol withdrawal syndrome. Mol. Biol. Rep..

[28] Schacht J.P., Hoffman M., Chen B.H., Anton R.F. (2022). Epigenetic moderators of naltrexone efficacy in reducing heavy drinking in Alcohol Use Disorder: A randomized trial. Pharmacogenom. J..

[29] Mons N., Beracochea D. (2016). Behavioral Neuroadaptation to Alcohol: From Glucocorticoids to Histone Acetylation. Front. Psychiatry.

[30] Warnault V., Darcq E., Levine A., Barak S., Ron D. (2013). Chromatin remodeling--a novel strategy to control excessive alcohol drinking. Transl. Psychiatry.

[31] Sharma R., Parikh M., Mishra V., Soni A., Rubi S., Sahota P., Thakkar M. (2022). Antisense-induced downregulation of major circadian genes modulates the expression of histone deacetylase-2 (HDAC-2) and CREB-binding protein (CBP) in the medial shell region of nucleus accumbens of mice exposed to chronic excessive alcohol consumption. J. Neurochem..

[32] Menger Y., Bettscheider M., Murgatroyd C., Spengler D. (2010). Sex differences in brain epigenetics. Epigenomics.

[33] McCarthy M.M., Nugent B.M. (2015). At the frontier of epigenetics of brain sex differences. Front. Behav. Neurosci..

[34] Jangra A., Sriram C.S., Pandey S., Choubey P., Rajput P., Saroha B., Bezbaruah B.K., Lahkar M. (2016). Epigenetic Modifications, Alcoholic Brain and Potential Drug Targets. Ann. Neurosci..

[35] Agudelo M., Figueroa G., Parira T., Yndart A., Muñoz K., Atluri V., Samikkannu T., Nair M.P. (2016). Profile of Class I Histone Deacetylases (HDAC) by Human Dendritic Cells after Alcohol Consumption and In Vitro Alcohol Treatment and Their Implication in Oxidative Stress: Role of HDAC Inhibitors Trichostatin A and Mocetinostat. PLoS ONE.

[36] Agudelo M., Gandhi N., Saiyed Z., Pichili V., Thangavel S., Khatavkar P., Yndart-Arias A., Nair M. (2011). Effects of alcohol on histone deacetylase 2 (HDAC2) and the neuroprotective role of trichostatin A (TSA). Alcohol Clin. Exp. Res..

[37] Licciardi P.V., Karagiannis T.C. (2012). Regulation of immune responses by histone deacetylase inhibitors. ISRN Hematol..

[38] Yu Y., Liu B., Chen S., Wang J., Chen F., Liu T., Jiang N., Chen W., Weng S., Cai X. (2022). Trichostatin A inhibits dendritic cell maturation through down-regulating NF-kappa B (p65) pathway. Mol. Biol. Rep..

[39] McBride W.J., Rodd Z.A., Bell R.L., Lumeng L., Li T.K. (2014). The alcohol-preferring (P) and high-alcohol-drinking (HAD) rats--animal models of alcoholism. Alcohol.

[40] Bell R.L., Hauser S.R., Liang T., Sari Y., Maldonado-Devincci A., Rodd Z.A. (2017). Rat animal models for screening medications to treat alcohol use disorders. Neuropharmacology.

[41] Bell R.L., Sable H.J., Colombo G., Hyytia P., Rodd Z.A., Lumeng L. (2012). Animal models for medications development targeting alcohol abuse using selectively bred rat lines: Neurobiological and pharmacological validity. Pharmacol. Biochem. Behav..

[42] Hauser S.R., Ferguson L.B., Liang T., Jarvis E.E., Mayfield R.D., Bell R.L. (2025). Effects of entinostat, quisinostat, and tubastatin-A on alcohol consumption in male high ethanol consuming rats. Front. Epigenet. Epigenom..

[43] Bell R.L., HS, Ferguson L.B., Manley A.L., Rodd Z.A., Mayfield R.D. (2020). Trichostatin A (TSA), an HDAC inhibitor, reduced EtOH intake in adult male and female HAD-1 rats. Alcohol Clin. Exp. Res..

[44] Marty V.N., Spigelman I. (2012). Effects of alcohol on the membrane excitability and synaptic transmission of medium spiny neurons in the nucleus accumbens. Alcohol.

[45] Sharma R., Mishra V., Parikh M., Soni A., Sahota P., Thakkar M. (2021). Antisense-induced knockdown of cAMP response element-binding protein downregulates Per1 gene expression in the shell region of nucleus accumbens resulting in reduced alcohol consumption in mice. Alcohol Clin. Exp. Res..

[46] Knapp C.M., Tozier L., Pak A., Ciraulo D.A., Kornetsky C. (2009). Deep brain stimulation of the nucleus accumbens reduces ethanol consumption in rats. Pharmacol. Biochem. Behav..

[47] Mulholland P.J., Padula A.E., Wilhelm L.J., Park B., Grant K.A., Ferguson B.M., Cervera-Juanes R. (2023). Cross-species epigenetic regulation of nucleus accumbens KCNN3 transcripts by excessive ethanol drinking. Transl. Psychiatry.

[48] Finn D.A., Hashimoto J.G., Cozzoli D.K., Helms M.L., Nipper M.A., Kaufman M.N., Wiren K.M., Guizzetti M. (2018). Binge Ethanol Drinking Produces Sexually Divergent and Distinct Changes in Nucleus Accumbens Signaling Cascades and Pathways in Adult C57BL/6J Mice. Front. Genet..

[49] Sakharkar A.J., Zhang H., Tang L., Baxstrom K., Shi G., Moonat S., Pandey S.C. (2014). Effects of histone deacetylase inhibitors on amygdaloid histone acetylation and neuropeptide Y expression: A role in anxiety-like and alcohol-drinking behaviours. Int. J. Neuropsychopharmacol..

[50] Bell R.L., Kimpel M.W., McClintick J.N., Strother W.N., Carr L.G., Liang T., Rodd Z.A., Mayfield R.D., Edenberg H.J., McBride W.J. (2009). Gene expression changes in the nucleus accumbens of alcohol-preferring rats following chronic ethanol consumption. Pharmacol. Biochem. Behav..

[51] Trapnell C., Williams B.A., Pertea G., Mortazavi A., Kwan G., van Baren M.J., Salzberg S.L., Wold B.J., Pachter L. (2010). Transcript assembly and quantification by RNA-Seq reveals unannotated transcripts and isoform switching during cell differentiation. Nat. Biotechnol..

[52] Anders S., Huber W. (2010). Differential expression analysis for sequence count data. Genome Biol..

[53] Anders S., Pyl P.T., Huber W. (2015). HTSeq--a Python framework to work with high-throughput sequencing data. Bioinformatics.

[54] Benjamini Y., Hochberg Y. (1995). Controlling the false discovery rate: A practical and powerful approach to multiple testing. J. R. Stat. Soc. B.

[55] Subramanian A., Tamayo P., Mootha V.K., Mukherjee S., Ebert B.L., Gillette M.A., Paulovich A., Pomeroy S.L., Golub T.R., Lander E.S. (2005). Gene set enrichment analysis: A knowledge-based approach for interpreting genome-wide expression profiles. Proc. Natl. Acad. Sci. USA.

[56] Mootha V.K., Lindgren C.M., Eriksson K.-F., Subramanian A., Sihag S., Lehar J., Puigserver P., Carlsson E., Ridderstråle M., Laurila E. (2003). PGC-1α-responsive genes involved in oxidative phosphorylation are coordinately downregulated in human diabetes. Nat. Genet..

[57] Liberzon A., Birger C., Thorvaldsdottir H., Ghandi M., Mesirov J.P., Tamayo P. (2015). The Molecular Signatures Database (MSigDB) hallmark gene set collection. Cell Syst..

[58] Chen T., Xu Y.P., Chen Y., Sun S., Yan Z.Z., Wang Y.H. (2023). Arc regulates brain damage and neuroinflammation via Sirt1 signaling following subarachnoid hemorrhage. Brain Res. Bull..

[59] Wang S., Zhang X., Zhao Y., Lv H., Li P., Zhang Z., Qiao X. (2024). BCI Improves Alcohol-Induced Cognitive and Emotional Impairments by Restoring pERK-BDNF. J. Mol. Neurosci..

[60] Liu Y.X., Wang J., Guo J., Wu J., Lieberman H.B., Yin Y. (2008). DUSP1 is controlled by p53 during the cellular response to oxidative stress. Mol. Cancer Res..

[61] Maurel O.M., Torrisi S.A., Barbagallo C., Purrello M., Salomone S., Drago F., Ragusa M., Leggio G.M. (2021). Dysregulation of miR-15a-5p, miR-497a-5p and miR-511-5p Is Associated with Modulation of BDNF and FKBP5 in Brain Areas of PTSD-Related Susceptible and Resilient Mice. Int. J. Mol. Sci..

[62] Van Looveren K., Van Boxelaere M., Callaerts-Vegh Z., Libert C. (2019). Cognitive dysfunction in mice lacking proper glucocorticoid receptor dimerization. PLoS ONE.

[63] Hirschey M.D., Shimazu T., Goetzman E., Jing E., Schwer B., Lombard D.B., Grueter C.A., Harris C., Biddinger S., Ilkayeva O.R. (2010). SIRT3 regulates mitochondrial fatty-acid oxidation by reversible enzyme deacetylation. Nature.

[64] Lee S.H., Lee J.H., Lee H.Y., Min K.J. (2019). Sirtuin signaling in cellular senescence and aging. BMB Rep..

[65] Verdin E., Hirschey M.D., Finley L.W., Haigis M.C. (2010). Sirtuin regulation of mitochondria: Energy production, apoptosis, and signaling. Trends Biochem. Sci..

[66] Graham D.L., Edwards S., Bachtell R.K., DiLeone R.J., Rios M., Self D.W. (2007). Dynamic BDNF activity in nucleus accumbens with cocaine use increases self-administration and relapse. Nat. Neurosci..

[67] Huang M.C., Schwandt M.L., Chester J.A., Kirchhoff A.M., Kao C.F., Liang T., Tapocik J.D., Ramchandani V.A., George D.T., Hodgkinson C.A. (2014). FKBP5 moderates alcohol withdrawal severity: Human genetic association and functional validation in knockout mice. Neuropsychopharmacology.

[68] Qiu B., Luczak S.E., Wall T.L., Kirchhoff A.M., Xu Y., Eng M.Y., Stewart R.B., Shou W., Boehm S.L., Chester J.A. (2016). The FKBP5 Gene Affects Alcohol Drinking in Knockout Mice and Is Implicated in Alcohol Drinking in Humans. Int. J. Mol. Sci..

[69] Qiu B., Xu Y., Wang J., Liu M., Dou L., Deng R., Wang C., Williams K.E., Stewart R.B., Xie Z. (2019). Loss of FKBP5 Affects Neuron Synaptic Plasticity: An Electrophysiology Insight. Neuroscience.

[70] Lein E.S., Hawrylycz M.J., Ao N., Ayres M., Bensinger A., Bernard A., Boe A.F., Boguski M.S., Brockway K.S., Byrnes E.J. (2007). Genome-wide atlas of gene expression in the adult mouse brain. Nature.

[71] Mili S., Moissoglu K., Macara I.G. (2008). Genome-wide screen reveals APC-associated RNAs enriched in cell protrusions. Nature.

[72] Stark K.L., Xu B., Bagchi A., Lai W.S., Liu H., Hsu R., Wan X., Pavlidis P., Mills A.A., Karayiorgou M. (2008). Altered brain microRNA biogenesis contributes to phenotypic deficits in a 22q11-deletion mouse model. Nat. Genet..

[73] Aston C., Jiang L., Sokolov B.P. (2005). Transcriptional profiling reveals evidence for signaling and oligodendroglial abnormalities in the temporal cortex from patients with major depressive disorder. Mol. Psychiatry.

[74] Sharma R., Chischolm A., Parikh M., Thakkar M. (2024). Cholinergic interneurons in the shell region of the nucleus accumbens regulate binge alcohol consumption: A chemogenetic and genetic lesion study. Alcohol Clin. Exp. Res..

[75] Solomon M.G., Nennig S.E., Cotton M.R., Whiting K.E., Fulenwider H.D., Schank J.R. (2024). Neurokinin-1 receptors in the nucleus accumbens shell influence sensitivity to social defeat stress and stress-induced alcohol consumption in male mice. Addict. Neurosci..

[76] Lepreux G., Henricks A.M., Wei G., Go B.S., Erikson C.M., Abella R.M., Pham A., Walker B.M. (2024). Kappa-opioid receptor antagonism in the nucleus accumbens shell distinguishes escalated alcohol consumption and negative affective-like behavior from physiological withdrawal in alcohol-dependence. Pharmacol. Biochem. Behav..

[77] Mitchell A.C., Mirnics K. (2012). Gene expression profiling of the brain: Pondering facts and fiction. Neurobiol. Dis..

[78] Denninger J.K., Walker L.A., Chen X., Turkoglu A., Pan A., Tapp Z., Senthilvelan S., Rindani R., Kokiko-Cochran O.N., Bundschuh R. (2022). Robust Transcriptional Profiling and Identification of Differentially Expressed Genes With Low Input RNA Sequencing of Adult Hippocampal Neural Stem and Progenitor Populations. Front. Mol. Neurosci..

[79] Liang T., Kimpel M.W., McClintick J.N., Skillman A.R., McCall K., Edenberg H.J., Carr L.G. (2010). Candidate genes for alcohol preference identified by expression profiling in alcohol-preferring and -nonpreferring reciprocal congenic rats. Genome Biol..

[80] Qiu S., Dong S., Fan J., Wu C., Qi X. (2025). Effect of high mobility group box 1 pathway inhibition on gene expression in the prefrontal cortex of mice exposed to alcohol. Alcohol.

[81] Mul J.D., Yi C.X., van den Berg S.A., Ruiter M., Toonen P.W., van der Elst M.C., Voshol P.J., Ellenbroek B.A., Kalsbeek A., la Fleur S.E. (2010). Pmch expression during early development is critical for normal energy homeostasis. Am. J. Physiol. Endocrinol. Metab..

[82] Ryabinin A.E., Fulenwider H.D. (2021). Alcohol and oxytocin: Scrutinizing the relationship. Neurosci. Biobehav. Rev..

[83] King C.E., Becker H.C. (2019). Oxytocin attenuates stress-induced reinstatement of alcohol seeking behavior in male and female mice. Psychopharmacology.

[84] King C.E., Griffin W.C., Lopez M.F., Becker H.C. (2021). Activation of hypothalamic oxytocin neurons reduces binge-like alcohol drinking through signaling at central oxytocin receptors. Neuropsychopharmacology.

[85] Peters S.T., Bowen M.T., Bohrer K., McGregor I.S., Neumann I.D. (2017). Oxytocin inhibits ethanol consumption and ethanol-induced dopamine release in the nucleus accumbens. Addict. Biol..

[86] Matsui S., Takahashi Y., Morioka S., Ozawa T., Kanayama S., Iwama H., Geng L., Umemoto K., Oguri Y., Tsuzuki S. (2026). Negative feedback regulation of alcohol ingestion through the FGF21-PVH oxytocin-VTA dopamine system. Proc. Natl. Acad. Sci. USA.

[87] Rodriguez K.M., Smith B.L., Caldwell H.K. (2020). Voluntary alcohol consumption is increased in female, but not male, oxytocin receptor knockout mice. Brain Behav..

[88] Lenz B., Weinland C., Bach P., Kiefer F., Grinevich V., Zoicas I., Kornhuber J., Muhle C. (2021). Oxytocin blood concentrations in alcohol use disorder: A cross-sectional, longitudinal, and sex-separated study. Eur. Neuropsychopharmacol. J. Eur. Coll. Neuropsychopharmacol..

[89] Potretzke S., Zhang Y., Li J., Fecteau K.M., Erikson D.W., Hibert M., Ryabinin A.E. (2023). Male-selective effects of oxytocin agonism on alcohol intake: Behavioral assessment in socially housed prairie voles and involvement of RAGE. Neuropsychopharmacology.

[90] Spence J.P., Reiter J.L., Qiu B., Gu H., Garcia D.K., Zhang L., Graves T., Williams K.E., Bice P.J., Zou Y. (2018). Estrogen-Dependent Upregulation of Adcyap1r1 Expression in Nucleus Accumbens Is Associated With Genetic Predisposition of Sex-Specific QTL for Alcohol Consumption on Rat Chromosome 4. Front. Genet..

[91] Quintana D.S., Glaser B.D., Kang H., Kildal E.S.M., Audunsdottir K., Sartorius A.M., Barth C. (2024). The interplay of oxytocin and sex hormones. Neurosci. Biobehav. Rev..

[92] Della Torre S., Maggi A. (2017). Sex Differences: A Resultant of an Evolutionary Pressure?. Cell Metab..

[93] Koyama Y., Nawa N., Ochi M., Surkan P.J., Fujiwara T. (2024). Epistatic interactions between oxytocin- and dopamine-related genes and trust. PLoS ONE.

[94] Muller S., Sicorello M., Moser D., Frach L., Limberg A., Gumpp A.M., Ramo-Fernandez L., Kohler-Dauner F., Fegert J.M., Waller C. (2023). The DNA methylation landscape of the human oxytocin receptor gene (OXTR): Data-driven clusters and their relation to gene expression and childhood adversity. Transl. Psychiatry.

[95] Li J., Li Y., Wang Z., Yao Y., Yao D., Chen K., Xia Y. (2025). Oxytocin Intervention Mitigates Pathological and Behavioral Impairments in APP/PS1 Mice Subjected to Early Social Isolation. CNS Neurosci. Ther..

[96] Selles M.C., Fortuna J.T.S., de Faria Y.P.R., Siqueira L.D., Lima-Filho R., Longo B.M., Froemke R.C., Chao M.V., Ferreira S.T. (2023). Oxytocin attenuates microglial activation and restores social and non-social memory in APP/PS1 Alzheimer model mice. iScience.

[97] Furman K.L., Baron L., Lyons H.C., Cha T., Evans J.R., Manna J., Zhu L., Mattis J., Burgess C.R. (2024). Melanin concentrating hormone projections to the nucleus accumbens enhance the reward value of food consumption and do not induce feeding or REM sleep. bioRxiv.

[98] Karlsson C., Rehman F., Damadzic R., Atkins A.L., Schank J.R., Gehlert D.R., Steensland P., Thorsell A., Heilig M. (2016). The melanin-concentrating hormone-1 receptor modulates alcohol-induced reward and DARPP-32 phosphorylation. Psychopharmacology.

[99] Lei K., Wegner S.A., Yu J.H., Mototake A., Hu B., Hopf F.W. (2016). Nucleus Accumbens Shell and mPFC but Not Insula Orexin-1 Receptors Promote Excessive Alcohol Drinking. Front. Neurosci..

[100] Karatayev O., Leibowitz S.F. (2025). Melanin-Concentrating Hormone (MCH): Role in Mediating Reward-Motivated and Emotional Behavior and the Behavioral Disturbances Produced by Repeated Exposure to Reward Substances. Int. J. Mol. Sci..

[101] Kuebler I.R.K., Suarez M., Wakabayashi K.T. (2024). Sex differences and sex-specific regulation of motivated behavior by Melanin-concentrating hormone: A short review. Biol. Sex Differ..

[102] Taniguchi M., Carreira M.B., Cooper Y.A., Bobadilla A.C., Heinsbroek J.A., Koike N., Larson E.B., Balmuth E.A., Hughes B.W., Penrod R.D. (2017). HDAC5 and Its Target Gene, Npas4, Function in the Nucleus Accumbens to Regulate Cocaine-Conditioned Behaviors. Neuron.

[103] McDonald A.J., Nemat P., van ‘t Hullenaar T., Schetters D., van Mourik Y., Alonso-Lozares I., De Vries T.J., Marchant N.J. (2024). Punishment-resistant alcohol intake is mediated by the nucleus accumbens shell in female rats. Neuropsychopharmacology.

[104] Schwarzman A.L., Gregori L., Vitek M.P., Lyubski S., Strittmatter W.J., Enghilde J.J., Bhasin R., Silverman J., Weisgraber K.H., Coyle P.K. (1994). Transthyretin sequesters amyloid beta protein and prevents amyloid formation. Proc. Natl. Acad. Sci. USA.

[105] Monaco H.L. (2000). The transthyretin-retinol-binding protein complex. Biochim. Biophys. Acta.

[106] Schreiber G. (2002). The evolutionary and integrative roles of transthyretin in thyroid hormone homeostasis. J. Endocrinol..

[107] Costa-Rodrigues D., Leite J.P., Saraiva M.J., Almeida M.R., Gales L. (2024). Transthyretin monomers: A new plasma biomarker for pre-symptomatic transthyretin-related amyloidosis. Amyloid.

[108] Richardson S.J., Wijayagunaratne R.C., D’Souza D.G., Darras V.M., Van Herck S.L. (2015). Transport of thyroid hormones via the choroid plexus into the brain: The roles of transthyretin and thyroid hormone transmembrane transporters. Front. Neurosci..

[109] Jono H., Su Y., Obayashi K., Tanaka Y., Ishiguro A., Nishimura H., Shinriki S., Ueda M., Ikeda K., Yamagata K. (2016). Sources of variation of transthyretin in healthy subjects in East and Southeast Asia: Clinical and experimental evidence for the effect of alcohol on transthyretin metabolism. Clin. Chim. Acta Int. J. Clin. Chem..

[110] Sun G., Peng Y., Hu C., Zheng Y., Cheng Y., Qi X., Jin Y. (2025). Alcohol exposure significantly influences gene expression in the hypothalamus, highlighting complex links with gonadotropin-releasing hormone signaling and thyroid hormone production in adolescent mice. BMC Med. Genom..

[111] Nixon P.F., Jordan L., Zimitat C., Rose S.E., Zelaya F. (2008). Choroid plexus dysfunction: The initial event in the pathogenesis of Wernicke’s encephalopathy and ethanol intoxication. Alcohol Clin. Exp. Res..

[112] McArdle C.J., Raab-Graham K.F. (2025). MicroRNA-mediated metabolic disruption as an emerging driver of alcohol use disorder. Nat. Commun..

[113] Xu C., Bai Q., Wang C., Meng Q., Gu Y., Wang Q., Xu W., Han Y., Qin Y., Jia S. (2020). miR-433 Inhibits Neuronal Growth and Promotes Autophagy in Mouse Hippocampal HT-22 Cell Line. Front. Pharmacol..

[114] Wang R., Zhang J. (2020). Clinical significance of miR-433 in the diagnosis of Alzheimer’s disease and its effect on Abeta-induced neurotoxicity by regulating JAK2. Exp. Gerontol..

[115] Ignacio C., Mooney S.M., Middleton F.A. (2014). Effects of Acute Prenatal Exposure to Ethanol on microRNA Expression are Ameliorated by Social Enrichment. Front. Pediatr..

[116] Huang B., Lang X., Li X. (2022). The role of IL-6/JAK2/STAT3 signaling pathway in cancers. Front. Oncol..

[117] Zhao X., Ma W., Li X., Li H., Li J., Li H., He F. (2021). ANXA1 enhances tumor proliferation and migration by regulating epithelial-mesenchymal transition and IL-6/JAK2/STAT3 pathway in papillary thyroid carcinoma. J. Cancer.

[118] Jones D.M., Read K.A., Oestreich K.J. (2020). Dynamic Roles for IL-2-STAT5 Signaling in Effector and Regulatory CD4(+) T Cell Populations. J. Immunol..

[119] Zhou B., Mei F., Wu C., Xu H., Liu Z., Cui Y. (2021). Protective effect of trichostatin A on CD19(+)CD5(+)CD1d(high) regulatory B cells in heart transplantation. Mol. Med. Rep..

[120] Yamamoto R., Saito M., Saito T., Sagehashi R., Koizumi A., Nara T., Kanda S., Numakura K., Narita S., Inoue T. (2020). Treg expansion with trichostatin A ameliorates kidney ischemia/reperfusion injury in mice by suppressing the expression of costimulatory molecules. Transpl. Immunol..

[121] Liran M., Fischer I., Elboim M., Rahamim N., Gordon T., Urshansky N., Assaf Y., Barak B., Barak S. (2025). Long-Term Excessive Alcohol Consumption Enhances Myelination in the Mouse Nucleus Accumbens. J. Neurosci..

[122] Murphy J.M., Stewart R.B., Bell R.L., Badia-Elder N.E., Carr L.G., McBride W.J., Lumeng L., Li T.K. (2002). Phenotypic and genotypic characterization of the Indiana University rat lines selectively bred for high and low alcohol preference. Behav. Genet..

[123] Soares A.R., Garcia-Rivas V., Fai C., Thomas M., Zheng X., Picciotto M.R., Mineur Y.S. (2025). Sex differences in the microglial response to stress and chronic alcohol exposure in mice. Biol. Sex Differ..

[124] Calvo-Enrique L., Lisa S., Vicente-Garcia C., Deogracias R., Arevalo J.C. (2023). Enhanced TrkA signaling impairs basal forebrain-dependent behavior. Front. Mol. Neurosci..

[125] Auld D.S., Mennicken F., Quirion R. (2001). Nerve growth factor rapidly induces prolonged acetylcholine release from cultured basal forebrain neurons: Differentiation between neuromodulatory and neurotrophic influences. J. Neurosci..

[126] Berse B., Lopez-Coviella I., Blusztajn J.K. (1999). Activation of TrkA by nerve growth factor upregulates expression of the cholinergic gene locus but attenuates the response to ciliary neurotrophic growth factor. Biochem. J..

[127] Zhou Z., Karlsson C., Liang T., Xiong W., Kimura M., Tapocik J.D., Yuan Q., Barbier E., Feng A., Flanigan M. (2013). Loss of metabotropic glutamate receptor 2 escalates alcohol consumption. Proc. Natl. Acad. Sci. USA.

[128] Jeon Y.J., Choi B.R., Park M.S., Jang Y.S., Yoon S., Lyoo I.K., Han J.S. (2023). Intact Recognition Memory and Altered Hippocampal Glucocorticoid Receptor Signaling in Fkbp5-deficient Mice Following Acute Uncontrollable Stress. Exp. Neurobiol..

[129] Touma C., Gassen N.C., Herrmann L., Cheung-Flynn J., Bull D.R., Ionescu I.A., Heinzmann J.M., Knapman A., Siebertz A., Depping A.M. (2011). FK506 binding protein 5 shapes stress responsiveness: Modulation of neuroendocrine reactivity and coping behavior. Biol. Psychiatry.

[130] Ferguson D., Koo J.W., Feng J., Heller E., Rabkin J., Heshmati M., Renthal W., Neve R., Liu X., Shao N. (2013). Essential role of SIRT1 signaling in the nucleus accumbens in cocaine and morphine action. J. Neurosci..

[131] Hao G., Mingchen Y., Yalin Z., Yaqi Q., Tingwu Y., Xinru X., Kaixuan L., Yani D., Dongsen L. (2025). Knockdown and overexpression of basolateral amygdala SIRT1 via AAV bidirectionally alter morphine-induced conditioned place preference extinction in mice. Front. Cell. Neurosci..

[132] Sharma S., Taliyan R., Ramagiri S. (2015). Histone deacetylase inhibitor, trichostatin A, improves learning and memory in high-fat diet-induced cognitive deficits in mice. J. Mol. Neurosci..

[133] Raghow R., Yellaturu C., Deng X., Park E.A., Elam M.B. (2008). SREBPs: The crossroads of physiological and pathological lipid homeostasis. Trends Endocrinol. Metab..

[134] Jang E.R., Lim S.J., Lee E.S., Jeong G., Kim T.Y., Bang Y.J., Lee J.S. (2004). The histone deacetylase inhibitor trichostatin A sensitizes estrogen receptor alpha-negative breast cancer cells to tamoxifen. Oncogene.

[135] Kim S.H., Kang H.J., Na H., Lee M.O. (2010). Trichostatin A enhances acetylation as well as protein stability of ERalpha through induction of p300 protein. Breast Cancer Res..

[136] McCarthy M.M., Auger A.P., Bale T.L., De Vries G.J., Dunn G.A., Forger N.G., Murray E.K., Nugent B.M., Schwarz J.M., Wilson M.E. (2009). The epigenetics of sex differences in the brain. J. Neurosci..

[137] Lee R.S., Tamashiro K.L., Yang X., Purcell R.H., Harvey A., Willour V.L., Huo Y., Rongione M., Wand G.S., Potash J.B. (2010). Chronic corticosterone exposure increases expression and decreases deoxyribonucleic acid methylation of Fkbp5 in mice. Endocrinology.

[138] Hartmann J., Wagner K.V., Liebl C., Scharf S.H., Wang X.D., Wolf M., Hausch F., Rein T., Schmidt U., Touma C. (2012). The involvement of FK506-binding protein 51 (FKBP5) in the behavioral and neuroendocrine effects of chronic social defeat stress. Neuropharmacology.

[139] Qiu B., Zhong Z., Righter S., Xu Y., Wang J., Deng R., Wang C., Williams K.E., Ma Y.Y., Tsechpenakis G. (2022). FKBP51 modulates hippocampal size and function in post-translational regulation of Parkin. Cell. Mol. Life Sci..

[140] Lieberman R., Kranzler H.R., Levine E.S., Covault J. (2017). Examining FKBP5 mRNA expression in human iPSC-derived neural cells. Psychiatry Res..

[141] Quintá H.R., Maschi D., Gomez-Sanchez C., Piwien-Pilipuk G., Galigniana M.D. (2010). Subcellular rearrangement of hsp90-binding immunophilins accompanies neuronal differentiation and neurite outgrowth. J. Neurochem..

[142] Williams K.E., Zou Y., Qiu B., Kono T., Guo C., Garcia D., Chen H., Graves T., Lai Z., Evans-Molina C. (2023). Sex-Specific Impact of Fkbp5 on Hippocampal Response to Acute Alcohol Injection: Involvement in Alterations of Metabolism-Related Pathways. Cells.

[143] Yong W., Eng M.Y., Shou W., Wall T.L., Liang T. (2011). Association of glucocorticoid receptor brinding protein Fkbp5 with alcohol drinking behavior in knockout mice and human subjects. Alcohol Clin. Exp. Res..

[144] Binder E.B., Salyakina D., Lichtner P., Wochnik G.M., Ising M., Putz B., Papiol S., Seaman S., Lucae S., Kohli M.A. (2004). Polymorphisms in FKBP5 are associated with increased recurrence of depressive episodes and rapid response to antidepressant treatment. Nat. Genet..

[145] Scharf S.H., Liebl C., Binder E.B., Schmidt M.V., Muller M.B. (2011). Expression and regulation of the Fkbp5 gene in the adult mouse brain. PLoS ONE.

[146] Basavarajappa B.S., Subbanna S. (2016). Epigenetic Mechanisms in Developmental Alcohol-Induced Neurobehavioral Deficits. Brain Sci..

[147] Zhou Z., Yuan Q., Mash D.C., Goldman D. (2011). Substance-specific and shared transcription and epigenetic changes in the human hippocampus chronically exposed to cocaine and alcohol. Proc. Natl. Acad. Sci. USA.

[148] Liu Y., Balaraman Y., Wang G., Nephew K.P., Zhou F.C. (2009). Alcohol exposure alters DNA methylation profiles in mouse embryos at early neurulation. Epigenetics.

[149] Dogan M.V., Lei M.K., Beach S.R., Brody G.H., Philibert R.A. (2016). Alcohol and tobacco consumption alter hypothalamic pituitary adrenal axis DNA methylation. Psychoneuroendocrinology.

[150] Kang J.I., Kim T.Y., Choi J.H., So H.S., Kim S.J. (2019). Allele-specific DNA methylation level of FKBP5 is associated with post-traumatic stress disorder. Psychoneuroendocrinology.

[151] Yang X., Ewald E.R., Huo Y., Tamashiro K.L., Salvatori R., Sawa A., Wand G.S., Lee R.S. (2012). Glucocorticoid-induced loss of DNA methylation in non-neuronal cells and potential involvement of DNMT1 in epigenetic regulation of Fkbp5. Biochem. Biophys. Res. Commun..

[152] Yeo S., Enoch M.A., Gorodetsky E., Akhtar L., Schuebel K., Roy A., Goldman D. (2017). The influence of FKBP5 genotype on expression of FKBP5 and other glucocorticoid-regulated genes, dependent on trauma exposure. Genes Brain Behav..

[153] Sabbagh J.J., O’Leary J.C., Blair L.J., Klengel T., Nordhues B.A., Fontaine S.N., Binder E.B., Dickey C.A. (2014). Age-associated epigenetic upregulation of the FKBP5 gene selectively impairs stress resiliency. PLoS ONE.

[154] Zannas A.S., Jia M., Hafner K., Baumert J., Wiechmann T., Pape J.C., Arloth J., Kodel M., Martinelli S., Roitman M. (2019). Epigenetic upregulation of FKBP5 by aging and stress contributes to NF-kappaB-driven inflammation and cardiovascular risk. Proc. Natl. Acad. Sci. USA.

[155] Paquette A.G., Lester B.M., Koestler D.C., Lesseur C., Armstrong D.A., Marsit C.J. (2014). Placental FKBP5 genetic and epigenetic variation is associated with infant neurobehavioral outcomes in the RICHS cohort. PLoS ONE.

[156] Gassen N.C., Fries G.R., Zannas A.S., Hartmann J., Zschocke J., Hafner K., Carrillo-Roa T., Steinbacher J., Preißinger S.N., Hoeijmakers L. (2015). Chaperoning epigenetics: FKBP51 decreases the activity of DNMT1 and mediates epigenetic effects of the antidepressant paroxetine. Sci. Signal..

[157] Calfa G., Chapleau C.A., Campbell S., Inoue T., Morse S.J., Lubin F.D., Pozzo-Miller L. (2012). HDAC activity is required for BDNF to increase quantal neurotransmitter release and dendritic spine density in CA1 pyramidal neurons. Hippocampus.

[158] Hodes G.E., Pfau M.L., Purushothaman I., Ahn H.F., Golden S.A., Christoffel D.J., Magida J., Brancato A., Takahashi A., Flanigan M.E. (2015). Sex Differences in Nucleus Accumbens Transcriptome Profiles Associated with Susceptibility versus Resilience to Subchronic Variable Stress. J. Neurosci..

