# Supplementary material for: Trichostatin A Modulates Ethanol Consumption and Reveals Dose- and Sex-Specific Transcriptomic Signatures in the Nucleus Accumbens Shell
Source: Cells. 2026 Aug 19;15(16):1494. doi: 10.3390/cells15161494 (PMC13511279; doi:10.3390/cells15161494)

Supplemental Table S1. The number of identified differentially expressed genes in males and females

Male						Female					
**1 mg/kg TSA vs. Control**						**1 mg/kg TSA vs. Control**					
Cutoff	FC > 1	FC > 1.5	FC > 2	FC > 3	FC > 4	Cutoff	FC > 1	FC > 1.5	FC > 2	FC > 3	FC > 4
p < 0.05	**247**	17	4	3	1	p < 0.05	**576**	27	4	2	1
p < 0.01	26	5	2	1	1	p < 0.01	160	20	4	2	1
**2 mg/kg TSA vs. Control**						**2 mg/kg TSA vs. Control**					
p < 0.05	**303**	27	0	0	0	p < 0.05	**163**	13	1	1	1
p < 0.01	58	11	0	0	0	p < 0.01	37	5	1	1	1
**2mg/kg vs. 1mg/kg TSA**						**2 mg/kg vs. 1mg/kg TSA**					
p < 0.05	**229**	24	5	1	1	p < 0.05	**81**	6	1	0	0
p < 0.01	67	13	4	1	1	p < 0.01	13	2	0	0	0

Table-S1: Number of identified differentially expressed genes between 1 mg/kg TSA vs. control; 2 mg/kg TSA vs. control; and between two dosages in males and females. Two levels of *p*-value cutoff and five different fold-change (FC) were applied.

Supplemental Table S2. Number of gene sets identified by GSEA.

	Na negative			Na positive		
**Male**	FDR<25%	FDR<5%	FDR<1%	FDR<25%	FDR<5%	FDR<1%
2 mg/kg	10	6	1	1031	345	102
1 mg/kg	1	0	0	534	14	3
**Female**						
2 mg/kg	416	0	0	0	0	0
1 mg/kg	691	72	0	21	1	0

Table-S2: Number of identified gene sets between 1 mg/kg TSA vs. control; 2 mg/kg TSA vs. control in both males and females using the different false discovery rate (FDR) cutoff.

Supplemental Table S3. Comparison of enrichment score of genes in oligodendrocyte markers gene set.

Oligodendrocyte markers				
Gene Name	Female 2 mg/kg Running ES	Female 1 mg/kg Running ES	Male 2 mg/kg Running ES	Male 1 mg/kg Running ES
CRYAB	**0.323**	**0.17**	-0.092	**0.506**
MTRR	**0.045**	-0.024	-0.059	**0.043**
MBP	**0.043**	-0.211	**0.161**	**0.22**
MAP2K6	**0.026**	**0.009**	-0.093	**0.02**
PCYT2	**0.021**	-0.056	**0.048**	**0.084**
SYT11	**0.001**	**0.032**	**0.011**	-0.075
SLC48A1	-0.004	-0.071	**0.106**	**0.142**
HCN2	-0.004	-0.127	**0.132**	**0.082**
TMBIM6	-0.006	**0.038**	**0.073**	**0.019**
EEF2	-0.006	**0.005**	**0.055**	**0.011**
PEX2	-0.01	-0.094	-0.027	**0.095**
TALDO1	-0.036	-0.032	**0.059**	**0.054**
RNF13	-0.036	-0.09	**0.012**	**0.077**
CD81	-0.039	-0.077	**0.058**	**0.017**
FABP5	-0.051	-0.067	**0.162**	**0.091**
NPC1	-0.056	-0.166	**0.155**	**0.181**
OMG	-0.059	-0.094	**0.034**	-0.031
CNTN2	-0.062	-0.161	**0.128**	**0.126**
TPPP3	-0.066	-0.013	**0.211**	**0.229**
AATK	-0.07	-0.133	**0.085**	**0.065**
ABCA2	-0.075	-0.19	**0.159**	**0.076**
SLC44A1	-0.08	-0.215	**0.095**	**0.211**
ARRDC3	-0.081	-0.027	**0.226**	**0.28**
NRBP2	-0.084	-0.113	**0.133**	**0.002**
APOD	-0.084	-0.342	**0.278**	**0.342**
MAL	-0.086	-0.256	**0.043**	**0.223**
HMGCS1	-0.105	-0.113	**0.022**	-0.07
GATM	-0.108	-0.196	**0.035**	**0.071**
MYRF	-0.11	-0.265	**0.164**	**0.205**
CSRP1	-0.111	-0.264	**0.197**	**0.229**
OLIG1	-0.114	-0.091	**0.116**	**0.176**
S100A16	-0.115	-0.166	**0.065**	**0.142**
SCD	-0.116	-0.164	**0.098**	**0.096**
SOX10	-0.12	-0.27	**0.149**	**0.166**
MOBP	-0.127	-0.267	**0.176**	**0.266**
CCP110	-0.136	-0.188	**0.01**	-0.035
PLEKHB1	-0.145	-0.241	**0.161**	**0.24**
WSCD1	-0.148	-0.242	**0.204**	**0.145**
S1PR5	-0.148	-0.303	**0.206**	**0.303**
CERS2	-0.163	-0.28	**0.199**	**0.123**
CLIC4	-0.163	-0.2	**0.107**	**0.191**
QDPR	-0.169	-0.333	**0.158**	**0.182**
MAG	-0.169	-0.351	**0.194**	**0.268**
MOG	-0.174	-0.375	**0.185**	**0.297**
CA2	-0.188	-0.311	**0.149**	**0.118**
NDRG1	-0.19	-0.3	**0.142**	**0.232**
TMEM63A	-0.193	-0.408	**0.14**	**0.183**
GJB1	-0.201	-0.451	**0.288**	**0.372**

FA2H	-0.215	-0.42	**0.282**	**0.223**
TMEM88B	-0.217	-0.37	**0.19**	**0.237**
PTGDS	-0.22	-0.34	**0.549**	**0.33**
GSN	-0.225	-0.339	**0.278**	**0.257**
UGT8	-0.226	-0.4	**0.123**	**0.228**
GLTP	-0.228	-0.34	**0.272**	**0.281**
CNP	-0.234	-0.4	**0.252**	**0.218**
ERMN	-0.247	-0.422	**0.14**	**0.17**
OLIG2	-0.271	-0.209	**0.21**	**0.153**
SERINC5	-0.281	-0.416	**0.33**	**0.198**
GPR37	-0.29	-0.481	**0.258**	**0.128**
SGK1	-0.302	-0.018	**0.288**	**0.25**
TSPAN2	-0.325	-0.477	**0.063**	**0.03**
MCAM	-0.413	-0.623	**0.42**	**0.199**
OPALIN	-0.429	-0.635	**0.382**	**0.157**
SERPINB1	-0.44	-0.204	**0.267**	-0.179

Table-S3: Alignment genes with their running enrichment score (ES) in oligodendrocyte markers gene set. Color highlights the positive (pink) or negative (blue) ES; a positive ES indicates enrichment among upregulated genes, while a negative ES indicates enrichment among downregulated genes. The color scheme depicts the different pattern of enrichment between males and females.

Supplemental Table S4. Hallmark Gene Sets

		NAME	NES	FDR q-val
Male- 2mg/kg vs Control	Na Positive	**Oligodendrocyte markers**	**2.6721075**	0.0000
		**Endothelium lymphatic vs blood dn**	**2.3700883**	0.0000
		**Neoplastic transformation CDC25 dn**	**2.3152955**	0.0000
		Liver cancer MYC E2F1 up	2.3125188	0.0000
		Bladder cancer cluster 2b	2.308278	0.0000
		Endothelium lymphatic vs blood up	2.3051398	0.0000
		Response to trabectedin up	2.2973716	0.0000
		Bladder cancer 2mg/kg recurrence	2.2807894	0.0000
		GLIS2 targets up	2.2768693	0.0000
		Angioimmunoblastic lymphoma up	2.2620718	0.0002
	Na Negative	**Pseudopodia haptotaxis up**	**-2.4182663**	0.0000
		Chronic hepatitis vs liver cancer up	-2.159071	0.0052
		SMARCA2 targets up	-1.9996625	0.0670
		Breast cancer progenitors up	-1.9972836	0.0517
		RNA degradation	-1.9660382	0.0683
Male-1mg/kg vs Control	Na Positive	**Oligodendrocyte markers**	**2.6817994**	0.0000
		**EZH2 targets up**	**2.3299518**	0.0016
		**Aging neocortex up**	**2.3100672**	0.0025
		Human parainfluenza virus 3 infection A594 cells dn	2.226222	0.0164
		Aging up	2.2228684	0.0142
		Aging brain up	2.2102194	0.0150
		Invasive carcinoma ductal vs lobular dn	2.199228	0.0166
		Response to trabectedin up	2.17	0.0240
		Dental caries up	2.17	0.0220
		MCV6 LCP with H3K4ME3	2.12	0.0460
	Na Negative	Non integrin membrane ECM interactions	-2.2807178	0.2878
		**Alzheimer's disease**	-2.2508888	**0.2264**
		All disorders CALB1 corr up	-2.1813858	0.3945
		MiR30 targets up	-2.1723976	0.3263
		Aging brain dn	-2.1700616	0.2662
		**NAME**	**NES**	**FDR q-val**
Female- 2mg/kg vs Control	Na Positive	Rapamycin sensitive genes	2.42	1.0000
		Voltage gated potassium channels	2	1.0000
		Cytosolic sulfonation of small molecules	1.96	1.0000
		Fc epsilon RI signaling pathway	1.9	1.0000
	Na Negative	**Oligodendrocyte markers**	-2.45	0.0000
		**Choroid plexus markers**	**-2.32**	0.0020
		**Aging up**	**-2.26**	0.0060
		Bladder cancer cluster 2b	-2.26	0.0050
		TRIM24 targets up	-2.24	0.0070
		SDHB targets 2	-2.2	0.0150
		**Response to TSA up**	**-2.17**	0.0310
		Response to leukotriene and thrombin	-2.16	0.0320
Female- 1mg/kg vs Control	Na Positive	**Prefrontal cortex 22q11 deletion up**	**2.15**	0.0060
		Neurexins and neuroligins	1.99	0.0640
		Localized to proximal dendrites	1.91	0.1500
	Na Negative	**Oligodendrocyte markers**	**-2.75**	**0.0000**
		Aging up	**-2.35**	**0.0000**
		**Major depressive disorder dn**	**-2.32**	0.0000
		Epithelial mesenchymal transition dn	-2.29	0.0000
		Aging neocortex up	-2.22	0.0010
		Response to TSA up	**-2.16**	0.0030
		Targets of HOXA9 and MEIS1 dn	-2.14	0.0040
		Resistance to neoplastic transfromation	-2.1	0.0090

Table-S4: Gene sets enriched in males and females administered both doses compared to the control. An Na positive gene set and positive normalized enrichment score (NES) indicates the gene set is at the

top of the ranked list. A Na negative gene set and negative NES score indicates the gene set is at the bottom of the list.

Supplemental Table S5. Genes enriched in myelin sheath cluster.

Genes Name	2 mg/kg vs control		1 mg/kg vs control		Gene Function	Function group
	Male	Female	Male	Female		
OPALIN	1.31	0.75	1.13	0.66	Mature oligodendrocyte marker; enriched in myelinating oligodendrocytes	Structure myelin gene
ERMN	1.12	0.84	1.17	0.75	Cytoskeletal protein important for oligodendrocyte process branching and sheath stability	
PLLP	1.29	0.85	1.23	0.73	Myelin membrane organization and lipid raft formation	
CNP	1.21	0.86	1.21	0.77	Early myelin protein; supports axon-glia interactions and myelin maintenance	
CLDN11	1.15	0.89	1.21	0.79	Tight-junction protein essential for compact CNS myelin	
UGT8	1.09	0.88	1.19	0.78	Generates galactocerebrosides, major myelin lipids	Myelin lipid synthesis genes
FA2H	1.25	0.87	1.18	0.78	Produces 2-hydroxylated fatty acids, critical for myelin membrane stability	
ADPGK	1.26	0.88	1.14	0.71	Glycolytic kinase supporting cellular energy metabolism	Oligodendrocyte metabolism
KLK6	1.28	0.86	1.31	0.74	Serine protease expressed in oligodendrocytes; involved in myelin remodeling and glial signaling	Extracellular remodeling

Table-S5: The genes enriched in myelin sheath cluster when comparing dosages in both males and females vs. control. Increasing gene expression was found in males but the opposite trend was found in females of both dosages, indicating different myelination modifications between sexes.

Supplemental Figure S1. Schematic diagram of testing procedure. Subjects were 60 days old at the start of the experiments, and the rats were allowed to consume 15% and 30% ethanol concurrently with water for eight weeks of 24-hr continuous access drinking. All rats received free access to standard laboratory chow and water throughout the experiments. Body weight (BW) and food intake (FI) were noted. The animals were maintained on a 12/12 hr reverse dark/light cycle (lights off at 1100 hrs). The rats were randomly assigned to control and dose groups (1 mg/kg and 2 mg/kg) during test week, and week 9 of ethanol drinking was used as a baseline. Brains were snap frozen followed by slicing and micro-punch on slides.

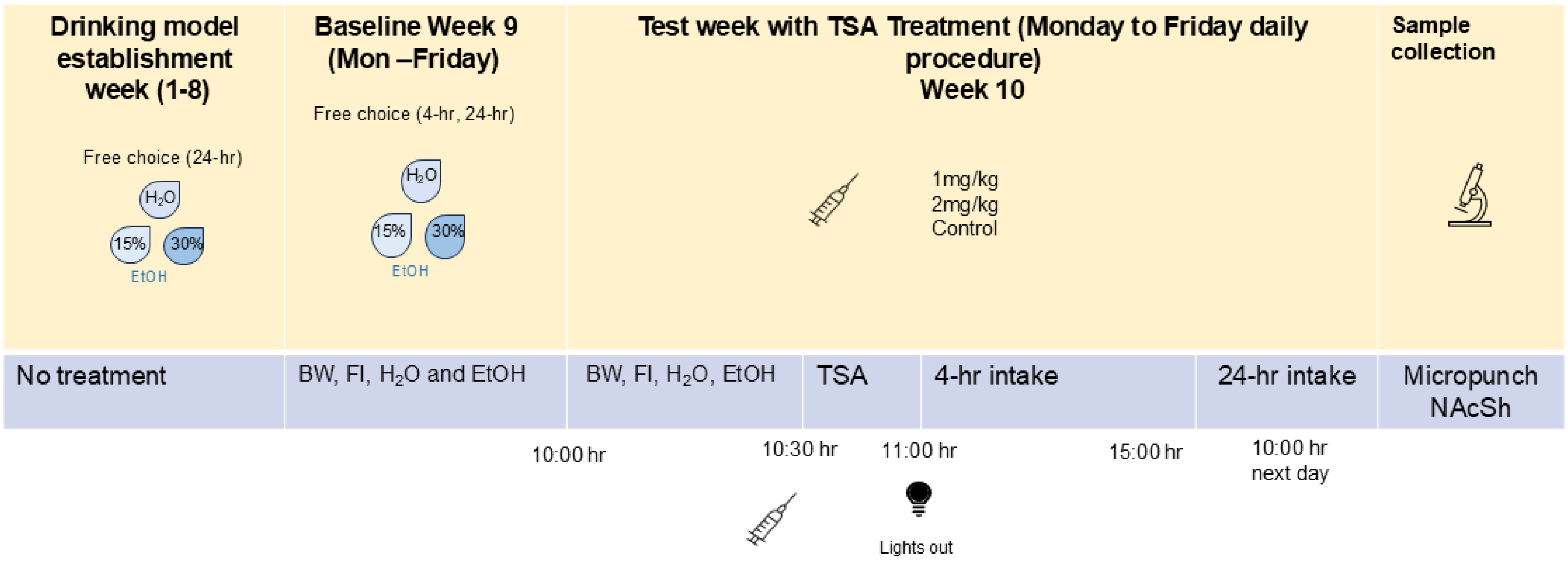

Supplemental Figure S2. IPA analysis identified prominent gene networks in male HAD1 rats after different dosages of TSA compared to the control. (A) Immune-function related gene network connected to NF-κB complex, IRF7 and neurotransmitter after 2 mg/kg dose of TSA. (B) Network involving FKBP5, TTR, and UBR1 is prominent in male rats administered a 1 mg/kg dose of TSA. Solid lines indicate direct physical interaction, and dashed lines indicate two molecules affecting each other through one or more intermediate or regulatory steps.

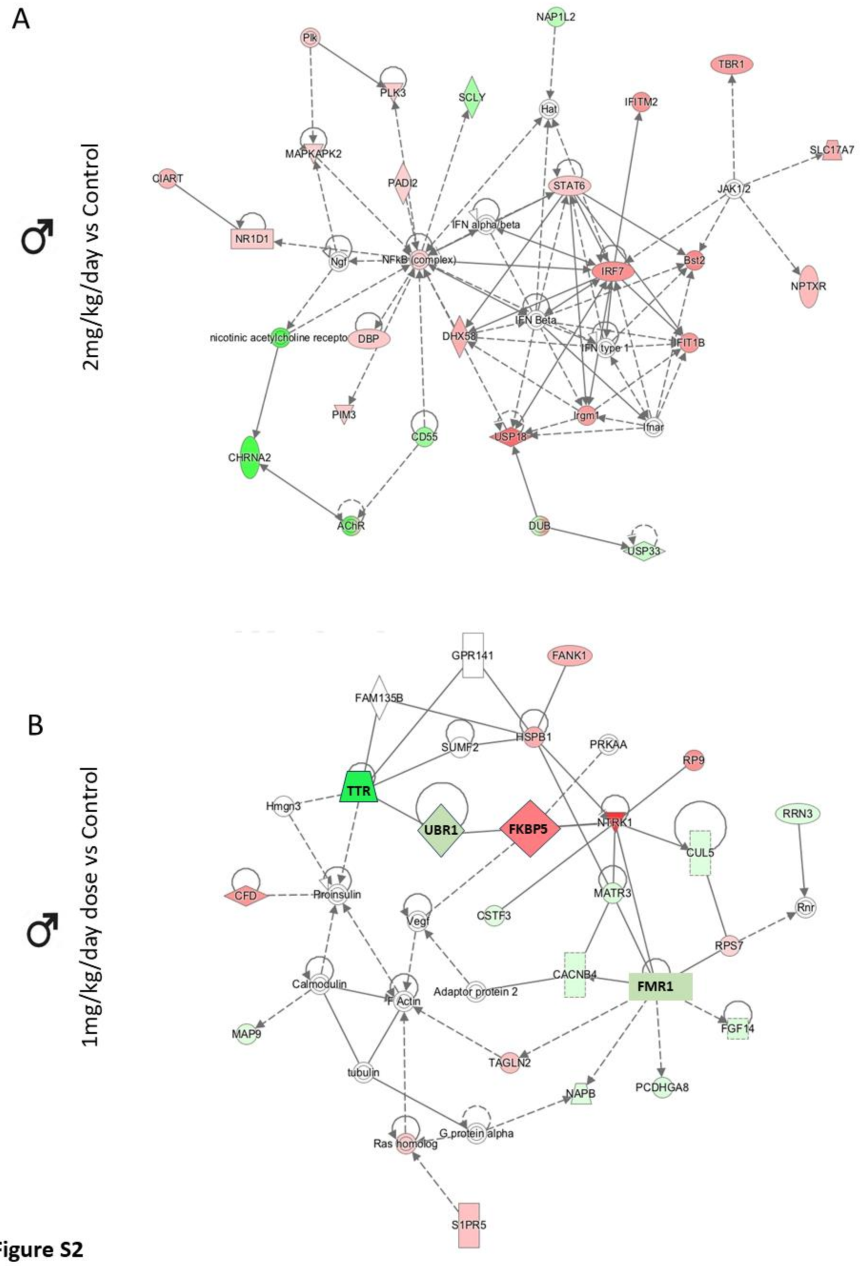

Figure S2

Supplemental Figure S3. Pathway alterations and genes differently impacted between 2 mg/kg and 1 mg/kg doses of TSA (A) in male and (B) in female HAD1 rats. Solid lines indicate direct physical interaction, and dashed lines indicate two molecules affecting each other through one or more intermediate or regulatory steps.

A Pathway alteration between dosage

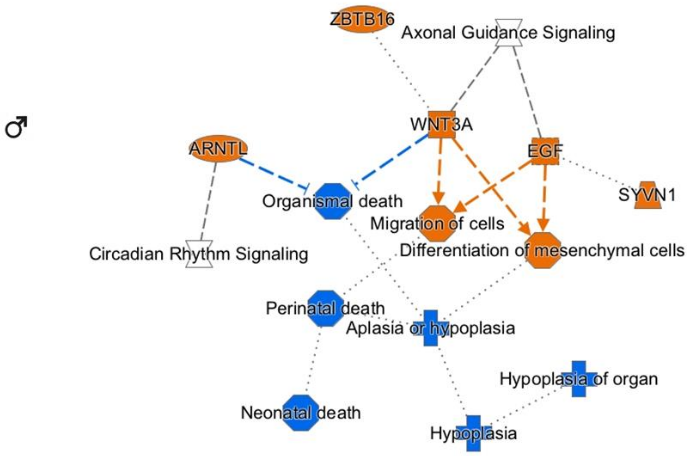

B Pathway alteration between dosage

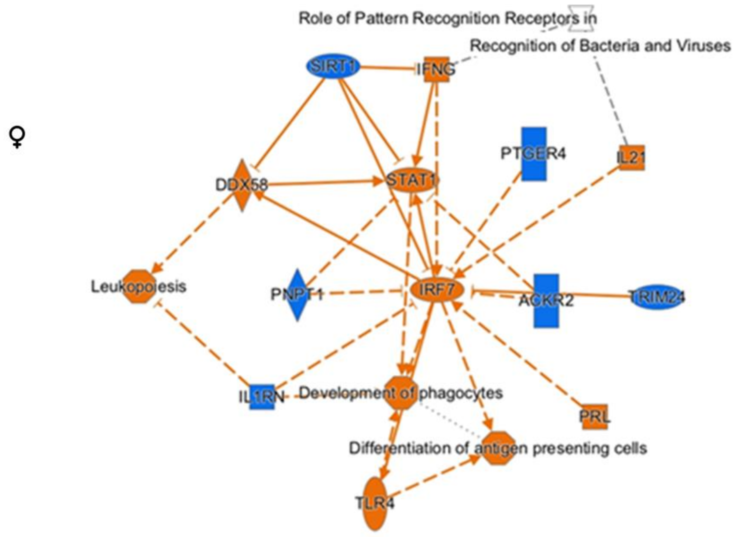

Figure S3

Supplemental Figure S4. Prominent gene networks in female HAD1 rats after different dosages of TSA compared to the control. (A) A gene network connecting BDNF to genes like Creb, NR4A1, and DUSP1 is significant in female rats administered a 2 mg/kg dose of TSA. (B) Network involving synaptic transmission, neuronal development and RNA process is prominent in female rats administered a 1 mg/kg dose of TSA. Green nodes indicate downregulated genes, and pink nodes indicate upregulated genes. Solid lines indicate direct physical interaction, and dashed lines indicate two molecules affecting each other through one or more intermediate or regulatory steps.

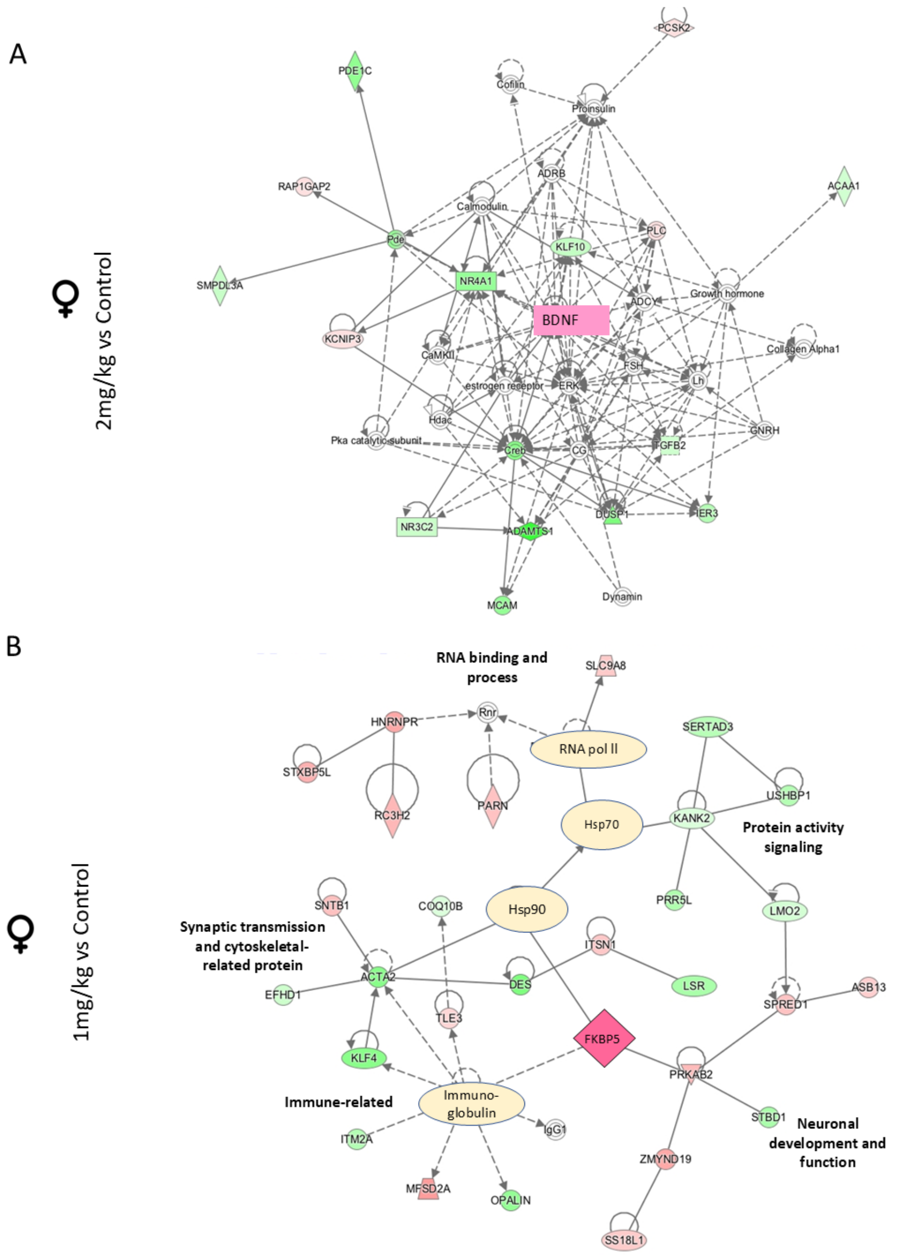

Figure S4

Supplemental Figure S5. Overview of GSEA enriched gene sets; each dot represents a gene set, and yellow highlighted areas are significant gene sets. (A) Male HAD1 rats after 2 mg/kg dosage TSA and (B) Male HAD1 rats after 1 mg/kg dosage TSA. (C) Female HAD1 rats after 2 mg/kg dosage TSA and (D) Female HAD1 rats after 1 mg/kg dosage TSA. A positive normalized enrichment score (NES) indicates gene set enrichment at the top of the ranked list; a negative ES indicates gene set enrichment at the bottom of the ranked list.

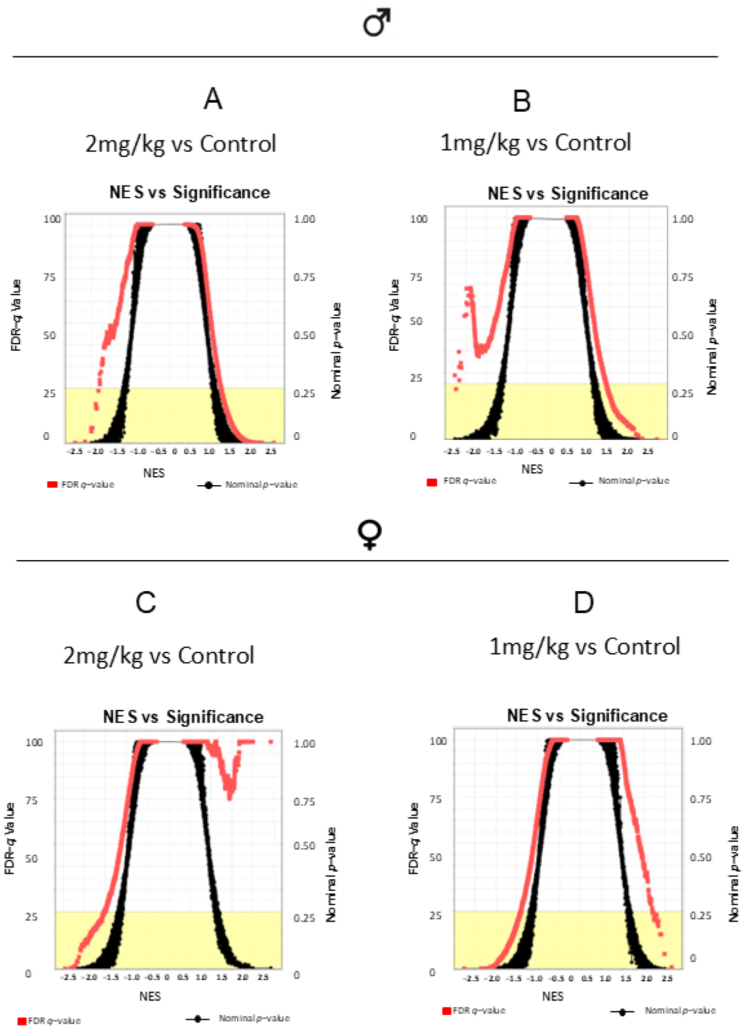

Supplemental Figure S6. Hallmark gene set analysis identified processes that are differentially enriched after a 2 mg/kg dose of TSA in (A) males and (B) females.

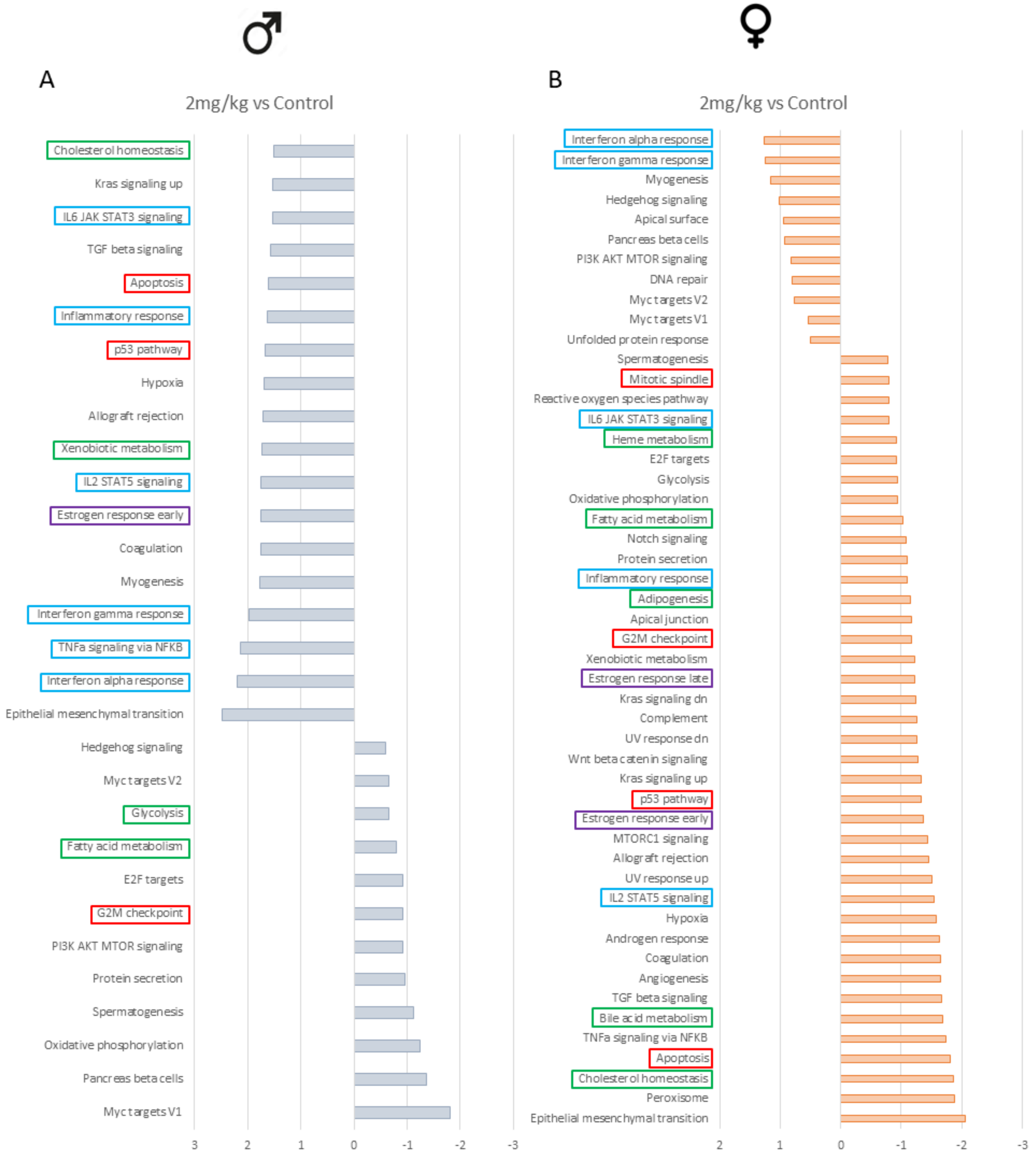

Supplemental Figure S7. Hallmark gene set analysis identified processes and functions that are differentially enriched after a 1 mg/kg dose of TSA in (A) males and (B) females.

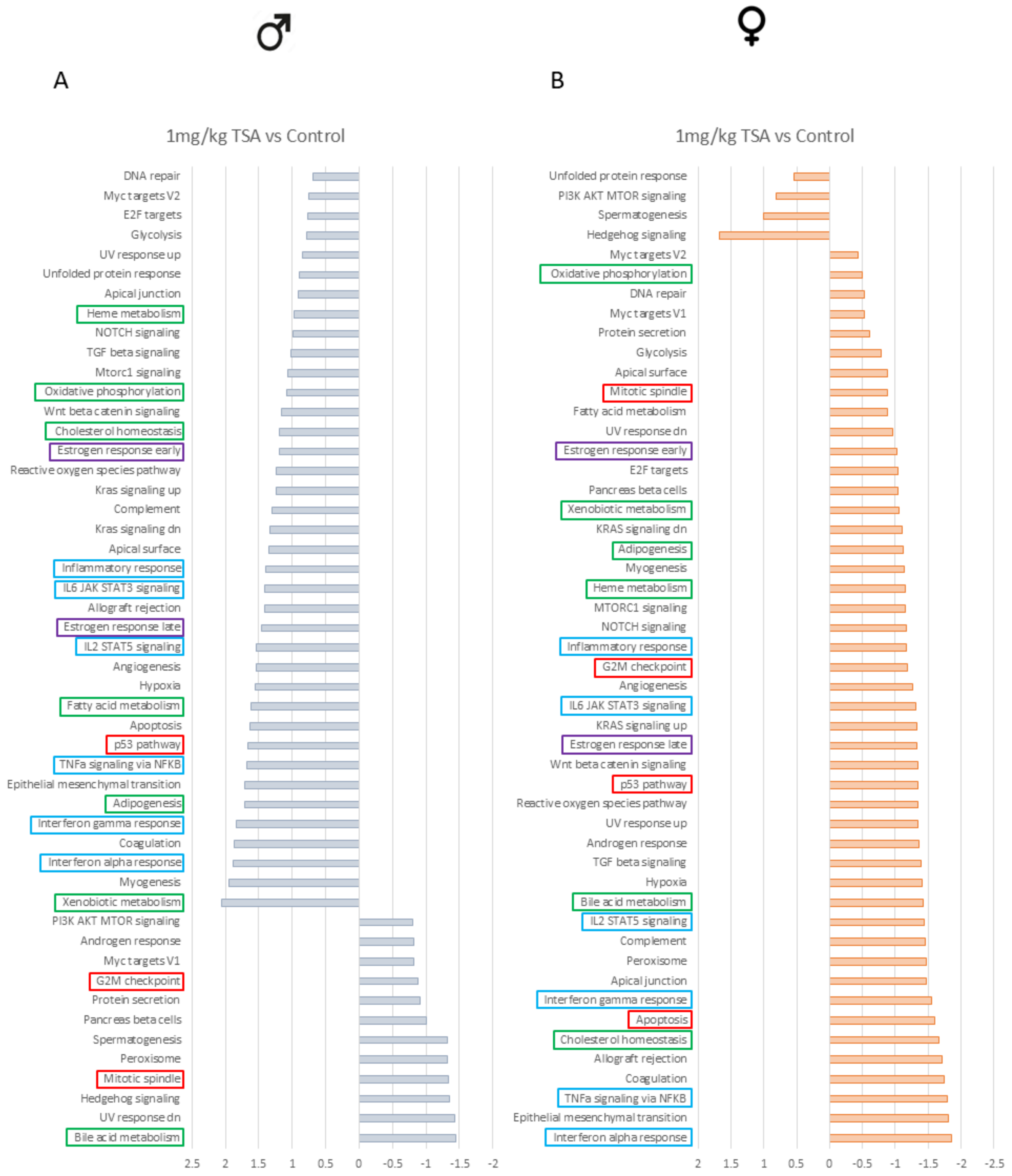

Supplement: Supplementary file 1 [file cells-15-01494-s001.zip › cells-4398709-supplementary.pdf]
